# SIS: a program to generate draft genome sequence scaffolds for prokaryotes

**DOI:** 10.1186/1471-2105-13-96

**Published:** 2012-05-14

**Authors:** Zanoni Dias, Ulisses Dias, João C Setubal

**Affiliations:** 1 Instituto de Computação, Universidade Estadual de Campinas, Campinas, SP, Brazil; 2 Departamento de Bioquímica, Instituto de Química, Universidade de São Paulo, São Paulo, SP, Brazil; 3 Virginia Bioinformatics Institute, Virginia Tech, Blacksburg, VA, USA

**Keywords:** Genome assembly, Contig order, Scaffold, Inversion, Prokaryotes

## Abstract

**Background:**

Decreasing costs of DNA sequencing have made prokaryotic draft genome sequences increasingly common. A contig scaffold is an ordering of contigs in the correct orientation. A scaffold can help genome comparisons and guide gap closure efforts. One popular technique for obtaining contig scaffolds is to map contigs onto a reference genome. However, rearrangements that may exist between the query and reference genomes may result in incorrect scaffolds, if these rearrangements are not taken into account. Large-scale inversions are common rearrangement events in prokaryotic genomes. Even in draft genomes it is possible to detect the presence of inversions given sufficient sequencing coverage and a sufficiently close reference genome.

**Results:**

We present a linear-time algorithm that can generate a set of contig scaffolds for a draft genome sequence represented in contigs given a reference genome. The algorithm is aimed at prokaryotic genomes and relies on the presence of matching sequence patterns between the query and reference genomes that can be interpreted as the result of large-scale inversions; we call these patterns inversion signatures. Our algorithm is capable of correctly generating a scaffold if at least one member of every inversion signature pair is present in contigs and no inversion signatures have been overwritten in evolution. The algorithm is also capable of generating scaffolds in the presence of any kind of inversion, even though in this general case there is no guarantee that all scaffolds in the scaffold set will be correct. We compare the performance of sis, the program that implements the algorithm, to seven other scaffold-generating programs. The results of our tests show that sis has overall better performance.

**Conclusions:**

sis is a new easy-to-use tool to generate contig scaffolds, available both as stand-alone and as a web server. The good performance of sis in our tests adds evidence that large-scale inversions are widespread in prokaryotic genomes.

## Background

With the decreasing costs of DNA sequencing it is now very common for prokaryotic genomes to be sequenced at “draft” status only. This means that the generated sequence will be a set of contigs (a contig is a substring of the string over the DNA alphabet that represents the genome sequence). The number of contigs depends on the sequencing fold coverage and DNA sequencing technology, and typically varies between half a dozen to a few hundred.

As of December 9, 2011, the number of draft microbial genome sequences in GenBank^a^ is 2324, compared to 1814 complete sequences. This difference is growing (in favor of draft sequences) over time (on March 15, 2011 the numbers were 1821 and 1485, respectively). Therefore there is an increasing need for tools that can improve the sequencing and assembly results beyond a simple contig set.

One technique that can be used to improve automated assembly results is to generate *contig scaffolds* from the contig set. A scaffold is an ordered set of contigs, with the desired order being the correct genome order, and with each contig in the correct orientation. Scaffolds help in whole genome comparisons and they can guide the finishing process, showing where the gaps are.

Some references in the literature adopt the term scaffold only for the situation where the contig ordering and orientation is given by paired-end read information [1]. Here we adopt a more general meaning, assuming a scaffold is a contig ordering obtained by any technique and/or additional data, or combination thereof.

There are various techniques that can help to create a scaffold. In addition to paired-end reads, one can use data from physical [2] or optical maps [3,4]. Information about the order of specific contigs sometimes can be obtained by searching for genes that have been split by the gaps between those contigs.

Another technique, which became popular in the last few years, is to use a reference genome. In this case we assume the query (draft) genome *A* has a close phylogenetic relative *B* that has been fully sequenced, and the genome of *B* can be used to guide the assembly of *A* and to generate a scaffold as well. This technique is used by programs such as ABACAS [5], fillScaffolds [6], Mauve Aligner [7], OSLay [8], Projector 2 [9], r2cat [10], PGA4genomics [11], and CONTIGuator [12].

When using a reference genome *B* to create a scaffold for *A*, one possible problem is the existence of rearrangements in *A* with respect to *B*. In prokaryotes, the most common rearrangement is the inversion [13], leading to the ‘X’ patterns that are frequently seen in dotplot graphs representing whole genome alignments of prokaryotic genomes [14] (when inversions are symmetric with respect to the origin of replication).

In this work we present an algorithm for obtaining contig scaffolds that explicitly takes into consideration the presence of inversions in *A* with respect to *B*. We do this by searching for *inversion signatures* in the input contigs. To our knowledge the only existing program that deals directly with inversions is fillScaffolds [6] (FillScaffolds deals with several other kinds of rearrangements besides inversions.). One other program seems to deal at least indirectly with rearrangements: Mauve Aligner [7], which is based on the multiple genome alignment program Mauve [15] and on GRIL [16].

We created program sis as an implementation of our algorithm, and tested it on real draft genomes that have been completed, comparing its performance to seven other programs. The criterion used to select genome sequences for testing was solely the availability of each genome in two formats: incomplete (several contigs) and complete (one contig per replicon). In these tests sis had the best overall performance.

## Methods

### Definitions

A *replicon* is a self-replication unit of a genome, such as a chromosome or a plasmid.

We represent a pair of single-replicon genomes by signed permutations, where numbers represent conserved genes or *blocks* between the two genomes, and the signs represent the plus or minus strand. We represent the reference genome by the identity permutation _*ιn*_=[ + 1, + 2, + 3,…, + *n*], and the query genome by the permutation *Π*=[_*Π*1_,_*Π*2_,_*Π*3_,…,_*Πn*_], where 1≤|_*Πi*_|≤*n*and |_*Πi*_|≠|_*Πj*_| if *i*≠*j*.

An inversion *ρ*(*i*,*j*) is a rearrangement event that reverses the order and the signs of a consecutive section of a genome: *Πρ*(*i*,*j*)=[_*Π*1_,…,_*Πi*−1_,−_*Πj*_,…,−_*Πi*_,_*Πj* + 1_,…,_*Πn*_], such that 1≤*i*≤*j*≤*n*. Two consecutive conserved blocks (_*Πi*_,_*Πi* + 1_) are a *breakpoint* if _*Πi*_≠_*Πi* + 1_−1; otherwise the consecutive blocks are an *adjacency*. A *strip* is a maximal substring [_*Πi*_,_*Πi* + 1_,…,_*Πj*_] such that every pair (_*Πk*_,_*Πk* + 1_) is an adjacency, for *i*≤*k*<*j*. We say that an inversion *ρ*(*r*,*s*)*acts* on a strip [_*Πi*_,_*Πi* + 1_,…,_*Πj*_] if *i*<*r*≤*s*<*j*.

**Figure 1:**
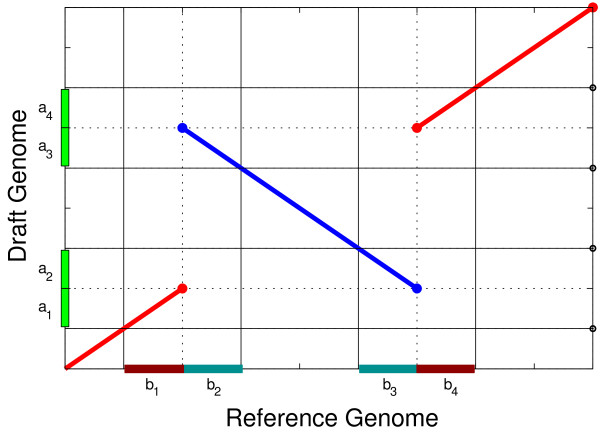
**Inversion Signatures.** The figure shows two regions with inversion signatures. The region *a*_1_–*a*_2_ in the draft genome matches regions *b*_1_ and *b*_3_ in the reference genome. *b*_1_ and *b*_3_ are not consecutive in the reference genome and hence this defines a breakpoint; moreover, because the match between *a*_1_ and *b*_1_ has a diffferent orientation as compared to the match between *a*_2_ and *b*_3_ this breakpoint is an inversion signature. The same happens with region *a*_3_–*a*_4_ of the draft genome and regions *b*_2_ and *b*_4_ of the reference genome. Both ISs are related because they are the result of the same inversion event. Therefore these two ISs constitute an IS pair.

An *inversion signature* (IS) is a breakpoint (_*Πi*__*Πi* + 1_) such that _*Πi*_ and _*Πi* + 1_ have different signs. Two ISs (_*Πi*__*Πi* + 1_) and (_*Πj*__*Πj* + 1_) are an *IS pair* if _*Πi*_=−_*Πj*_−1 and _*Πi* + 1_=−_*Πj* + 1_ + 1 (see Figure 1); our concept of inversion signature is unrelated to a concept of similar name presented in [17].

**Figure 2:**
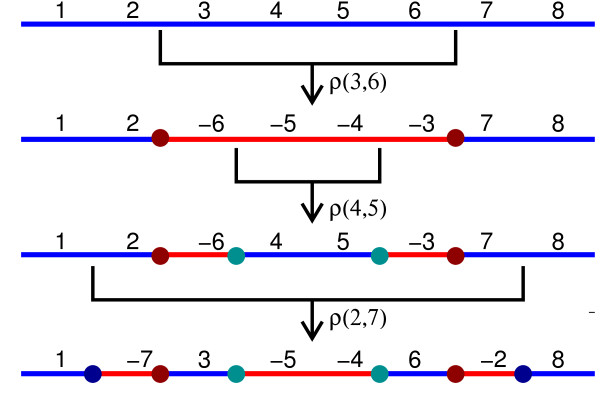
**Symmetric Inversions.** Three symmetric inversions *ρ*(3,6), *ρ*(4,5) and *ρ*(2,7) applied to the identity permutation _*ι*8_. The final result does not depend on the order of the inversions. The colored circles represent IS pairs created by the inversions.

**Figure 3:**
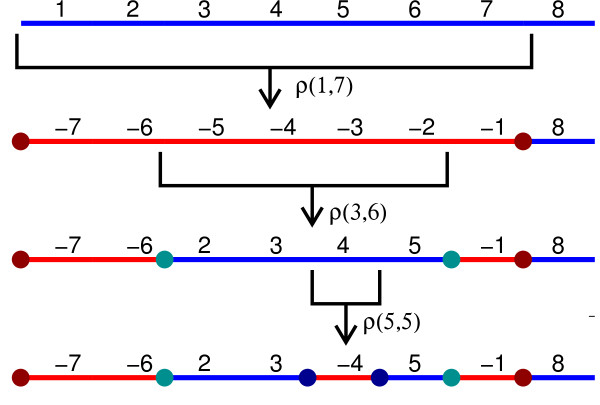
**Nested Inversions.** Three nested inversions *ρ*(1,7), *ρ*(3,6) and *ρ*(5,5) applied to the identity permutation _*ι*8_. The final result may change if the order of the inversions changes. The colored circles represent IS pairs created by the inversions.

**Figure 4:**
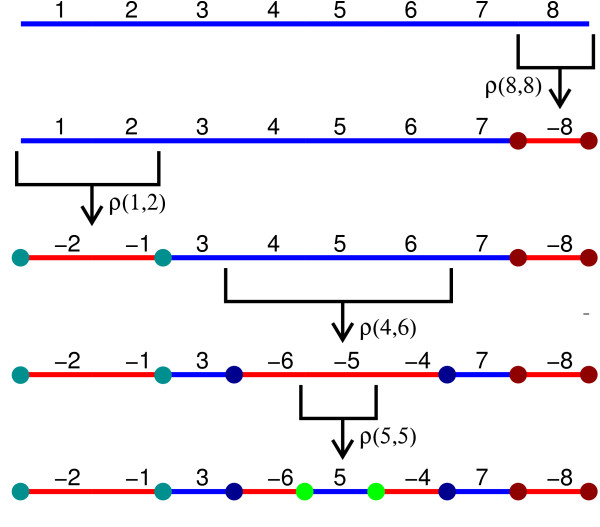
**Safe Inversions.** Four safe inversions *ρ*(8,8), *ρ*(1,2), *ρ*(4,6) and *ρ*(5,5) applied to the identity permutation *ι*_8_. The colored circles represent IS pairs created by the inversions.

**Figure 5:**
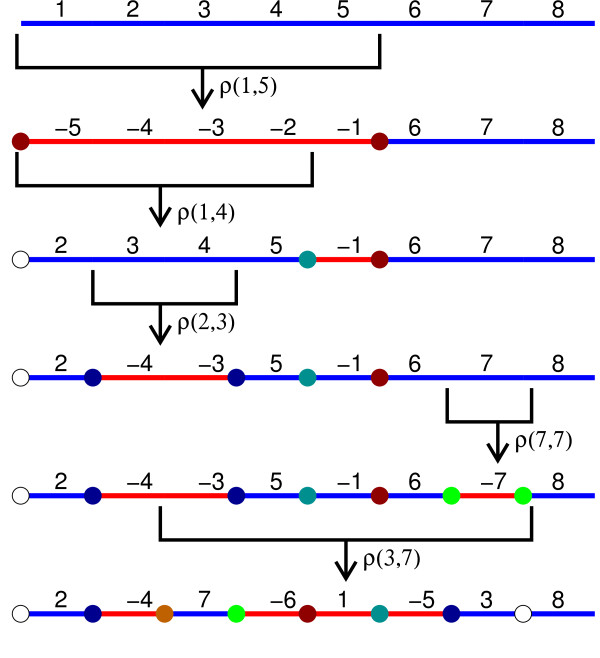
**Generic Inversions.** Five generic inversions *ρ*(1,5), *ρ*(1,4), *ρ*(2,3), *ρ*(7,7) and *ρ*(3,7) applied to the identity permutation *ι*_8_. The white circles represent ISs overwritten by nonsafe inversions.

An inversion *ρ*(*i*,*j*) is *symmetric* if *n*=*i* + *j*−1 (Figure 2). An inversion *ρ*(_*i*1_,_*j*1_) is *nested* with respect to an inversion *ρ*(_*i*2_,_*j*2_) if _*i*2_<_*i*1_≤_*j*1_<_*j*2_ (Figure 3). An inversion *ρ*(*i*,*j*) is *safe* with respect to *Π*if *ρ*(*i*,*j*) acts on a strip [_*Πr*_,_*Πr* + 1_,…,_*Πs*_] of *Π* (Figure 4). An inversion *ρ*(*i*,*j*) without any restrictions on the values of *i* and *j* is a *generic inversion* (Figure 5).

Any sequence of symmetric inversions can be sorted so that the result is a series of nested inversions, with the same effect on a genome. Every series of nested inversions is a series of safe inversions, but the converse is not true.

Let _*P*1_(*n*), _*P*2_(*n*), _*P*3_(*n*) and _*P*4_(*n*) denote the sets of all permutations that can be obtained from _*ιn*_by applying a series of symmetric inversions, a series of nested inversions, a series of safe inversions, and a series of generic inversions, respectively. Clearly, $P_{1}(n)\subset P_{2}(n)\subset P_{3}(n)\subset P_{4}(n)$.

A collection of *m* contigs from a genome *Π* is represented by a collection of substrings *C*_1_, *C*_2_, …, *C*_m_, such that, for 1≤*k*≤*m*, $C_{k}=[\Pi_{i_{k}},\ldots ,\Pi_{j_{k}}]$ or $C_{k}=[-\Pi_{j_{k}},\ldots ,-\Pi_{i_{k}}]$, and the intervals in *Π* that correspond to any two contigs $C_{k_{1}}$ and $C_{k_{2}}$ cannot have an overlap, that is $[i_{k_{1}}\mathrm{..}j_{k_{1}}]\cap [i_{k_{2}}\mathrm{..}j_{k_{2}}]=\emptyset$, for 1≤_*k*1_<_*k*2_≤*m*.

### Algorithm

The algorithm we have developed generates correct scaffolds in the presence of symmetric, nested, or safe inversions (as long as at least one IS is present for every IS pair); in addition it can also produce scaffolds, not necessarily 100% correct, for generic inversions.

The algorithm depends on the following assumptions in order to generate one single correct scaffold: (1) There are no duplicated conserved blocks in either of the two genomes; (2) all contigs are correctly assembled; (3) both genomes contain only one chromosome; and (4) conserved block 01 is in the first position (with + sign) or in the last position (with - sign), in some contig.

Assumption (1) means that there is no conserved block that contains a sequence that is a repeat in any of the two genomes. This is somewhat unrealistic, since all genomes in different degrees have repeats. In this sense, assumption (2) also depends on the lack of repeats. But as the tests below show, these assumptions do not seem to be major obstacles. In particular, repeats account for no more than 13% of errors in contig adjacencies in our tests. We require assumption (3) primarily for simplicity of exposition; in a real setting it is generally possible to decompose scaffold creation for separate replicons as separate problems. Assumption (4) helps simplify the algorithm description. In case block 01 does not follow assumption (4), a remapping of conserved blocks can be made in time *O*(*n*) such that the result does follow assumption (4).

The algorithm can detect lack of information, caused by absence of an IS pair in the input or by the overwriting of an earlier (in evolution) IS by a later unsafe inversion, and still generate a scaffold. In such cases the algorithm chooses a contig that has not been positioned yet, and begins generation of a new scaffold (so the result will be a set of scaffolds). When IS pairs are missing there are no guarantees that the scaffolds generated are correct.

The input for the algorithm is a list of contigs, each contig represented by the conserved blocks it contains. Note that we deal only with conserved blocks; insertions and deletions of the query genome with respect to the reference genome are not considered.

We now give a detailed example to illustrate the various concepts as well as to present the algorithm. In this example, there are 40 conserved blocks distributed in 9 contigs as follows: 

· [−23,−22, + 19, + 20]

· [−15,−14, + 12, + 13,−11]

· [ + 34, + 35]

· [ + 36, + 37, + 38, + 39, + 40]

· [−03,−02,−01]

· [ + 05, + 06, + 07, + 08]

· [ + 31, + 32, + 33,−30,−29]

· [−28,−27, + 26,−25,−24, + 04]

· [ + 21,−18,−17,−16, + 09, + 10]

The correct order for the blocks in the query genome is given as follows:

(1) $$\begin{array}{cc} \Pi = & [+01,+02,+03,-23,-22,+19,+20,+21,\\ \;-18,-17,-16,+09,+10,+11,-13,-12,\\ \;+14,+15,-08,-07,-06,-05,-04,+24,\\ \;+25,-26,+27,+28,-35,-34,+31,+32,\\ \;+33,-30,-29,+36,+37,+38,+39,+40] \end{array}$$

Recall that the reference genome is the identity permutation. The IS pairs are the following: 

· [ + 03,−23] × [−04, + 24]

· [−22, + 19] × [ + 21,−18]

· [−16, + 09] × [ + 15,−08]

· [ + 11,−13] × [−12, + 14]

· [ + 25,−26] × [−26, + 27]

· [ + 28,−35] × [−29, + 36]

· [−34, + 31] × [ + 33,−30]

An inspection reveals that the query genome has 7 safe inversions with respect to the reference genome. However, note that only the following ISs are present in the contigs: 

· × [−04, + 24]

· [−22, + 19] × [ + 21,−18]

· [−16, + 09] ×

· [ + 11,−13] × [−12, + 14]

· [ + 25,−26] × [−26, + 27]

· × [ + 33,−30]

Note that IS pair 6 is missing from the input contigs.

The algorithm builds the scaffold incrementally, choosing one contig following certain rules and placing it in the scaffold immediately after the previously chosen contig. The first contig chosen is the one that contains block +01 (or -01).

#### Execution of the algorithm on the example

**Figure 6:**
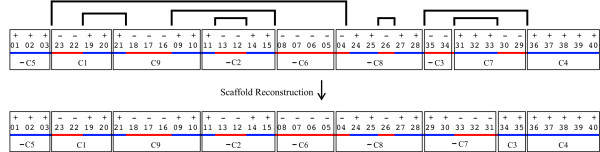
**Scaffold Generated in the Presence of Safe Inversions.** Illustration of the example discussed in the text of a genome with 40 conserved blocks with respect to a reference genome. The query genome and the reference genome differ by seven safe inversions, denoted by the nested horizontal square brackets. The conserved blocks appear in nine contigs. The permutation on the top is the “real” query genome, and the permutation at the bottom is the scaffold reconstructed by the algorithm. The bars with alternating colors help visualize inversion signatures.

In the example, block 01 is found inverted in the last position of contig 5 (see Figure 6). Thus, the algorithm reverse complements contig 5 and inserts it as the first component of the scaffold. The next conserved block searched is + 04, which should be in the first position of its contig on the plus strand or in the last position of its contig on the minus strand. Block 04 is found in the last position of contig 8 on the minus strand, which is interpreted by the algorithm to be the result of a safe inversion. The algorithm then uses the IS (−24, + 04) to search for block −23, which should be at the other end of the inversion that created the IS found. Block −23 is found in the first position of contig 1, so this contig becomes the second in the scaffold.

The next conserved block to be searched is + 21. It is found at the beginning of contig 9, on the plus strand as expected, so this contig is placed in the third scaffold position. The next block searched is + 11, which is found inverted in the last position of contig 2. This means that contig 2 will be inserted in the fourth scaffold position after it is reverse-complemented. The next block to be searched is + 16, which is found on the minus strand in the middle of the already placed contig 9. The algorithm then uses the IS (−16, + 09) to determine that the next block to be searched is −08. Block −08 is found on the plus strand in the last position of contig 6. The subsequent steps proceed along the same lines. The resconstructed scaffold is [−_*C*5_,−_*C*1_, + _*C*9_,−_*C*2_,−_*C*6_,−_*C*8_,−_*C*7_, + _*C*3_, + _*C*4_]. This scaffold contains errors, because the correct scaffold given by permutation *Π* is [−_*C*5_,−_*C*1_, + _*C*9_,−_*C*2_,−_*C*6_,−_*C*8_,−_*C*3_, + _*C*7_, + _*C*4_]. This was caused by the fact that from the given input it is impossible to tell that contig 3 should remain as it is and that contig 7 should be reverse complemented. This stems from the fact that the endpoints of IS pair 6 are not to be found in the input contigs. But the algorithm still managed to get 7 correct adjacencies out of 9.

**Figure 7:**
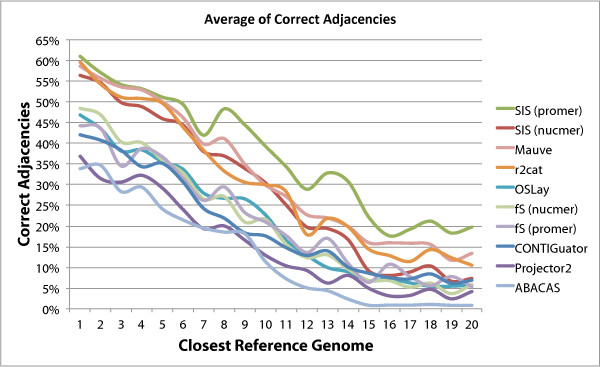
**Algorithm to determine a genome scaffold based on inversion signatures.** Data structures used are explained in the text.

Pseudocode for the algorithm is given in Figure 7. The algorithm assumes that the following data structures are available and filled in: contigs stores contig information and inContig contains conserved block information, storing for each conserved block the position in structure contigs where it can be found, as well as the contig number and the sign (strand). More formally, if contigs [*c*][*p*]=*x*, then inContig [|*x*|].contig=*c*, inContig [|*x*|].pos=*p* and inContig[|*x*|]. *x*=*s*, such that *s*×|*x*|=*x*. For example, contigs[7] =[ + 31, + 32, + 33,−30,−29], contigs[7][4] =−30, inContig[|-30|].contig = 7, inContig [|-30|].pos = 4 and inContig [|-30|].sign =−1.

In addition to the data structures described above the algorithm uses two auxiliary vectors: used indicates which contigs have been placed in the scaffold; and searched records conserved blocks that have been searched so far. Variable nextC indicates the contig with smallest ID not yet placed in the scaffold.

The main loop in lines 7–39 is executed until all contigs have been placed in the scaffold. Variable ok indicates whether the algorithm found a candidate contig to be placed in the scaffold. Line 9 ensures that the algorithm is not searching for an invalid conserved block. Lines 21–24 check whether the algorithm is searching an already searched conserved block.

If the algorithm decides to insert a new contig in the scaffold (test in line 25) lines 26–39 are executed. If no valid contig was found, the first contig not yet placed is determined in lines 26–28. Lines 29–30 place that contig as the first in a new scaffold. Vector used and variables nextC and i are updated in lines 31–34.

Recalling that *n* is the number of conserved blocks and *m* is the number of contigs, with *n*≥*m*, we now state the algorithm’s complexity. Data structure inContig can be built in *O*(*n*). Initialization takes *O*(*n* + *m*). The main loop is executed *n* + *m* times, since in each iteration either a new position in vector searched is marked or a new contig is placed in the scaffold. There is one operation inside the main loop that does not take constant time, which is the loop in lines 33–34. However, variable nextC can be incremented at most *m* times, so the amortized cost of each update is *O*(1). This leads to an overall complexity of *O*(*n*).

### Testing Methodology

We have implemented the algorithm creating program sis (Scaffolds from Inversion Signatures). We stress that sis does not require the algorithm assumptions listed above in order to be used. We used real contigs available in GenBank for testing as described below.

The main quality measure for a scaffold is the number of correct contig adjacencies. A contig adjacency for contigs *x* and *y* is correct if they are consecutive in the (completed) query genome as well as in the generated scaffold set. In the case of circular replicons, all correct scaffolds with *m* contigs have exactly *m* correct adjacencies. Even though SIS in general outputs a set of scaffolds, for the purposes of this test we consider the result to be just one scaffold, with scaffolds being placed side by side as the algorithm creates them.

A second quality measure is genome coverage. In this measure we attempt to determine how much of the genome to which the contigs belong is actually covered by the scaffolds generated. The working definition we use is as follows. If both ends of a contig have correct adjacencies, then we count the entire length of that contig as contributing to the coverage. If only one end has a correct adjacency, then half of the contig length is used. If both ends have incorrect adjacencies, then the contig is not used. We define coverage as the ratio of the sum of contig lengths considered per the above rules and the sum of all contig lengths.

In this test we compared sis to seven other scaffold generating programs, namely ABACAS [5], fillScaffolds [6], Mauve Aligner [7], OSLay [8], Projector 2 [9], r2cat [10], and CONTIGuator [12]. Table 1 gives a summary of all programs tested.

**Table 1:** Programs Tested


	**Prokaryote vs.**	**Unichromosomal vs.**	**Standalone**	**Block**	**User**
	**Eukaryote**	**Multichromosomal**	**vs. Server**	**Detection**	**Interface**
**Abacas**	Prokaryote	Multichromosomal	Standalone	nucmer, promer	Command line
**CONTIGuator**	Prokaryote	Multichromosomal	Standalone	Blast+	Command line
**fillScaffolds**	Eukaryote	Multichromosomal	Standalone	nucmer, promer	Command line
**Mauve Aligner**	Prokaryote	Unichromosomal	Standalone	Mauve	GUI
**OSLay**	Both	Unichromosomal	Standalone	nucmer	GUI
**Projector2**	Prokaryote	Unichromosomal	Server	BLAT	Web
**r2cat**	Both	Multichromosomal	Standalone	-	GUI
**SIS**	Prokaryote	Unichromosomal	Both	nucmer, promer	Web, Command line

It is important to mention that among programs tested fillScaffolds [6] was the only one designed specifically for eukaryote sequences, whereas all of our tests are done on prokaryote sequences. FillScaffolds is a sophisticated tool that takes into account many kinds of rearrangements in addition to inversions, such as transpositions, reciprocal translocations, and chromosome fusions and fissions. We have included fillScaffolds in the present study for completeness.

One important consideration is block conservation detection. In the case of sis this was done by programs nucmer [18] and promer [18], with default settings. We postprocessed the outputs using the MUMmer script delta-filter [18] with parameter −1 , which ensures that only nonrepeated blocks are processed.

Mauve Aligner uses progressiveMauve [15] for sequence comparison. OSLay can use nucmer and that is what we chose (with default settings). r2cat does sequence comparison internally. Projector 2 can use BLAT [19] (default) or BLAST [20], and we chose BLAT. CONTIGuator uses BLAST+ [21]. ABACAS can use nucmer or promer. We chose nucmer. (We observed that choosing promer made ABACAS run for more than two days without providing any scaffold.) For fillScaffolds [6] we have used both nucmer and promer.

#### Input genomes

We have tested the programs on real contigs from preliminary assemblies of 19 genomes. These genomes have been finished, so that we know the correct order for contigs in all cases. The list of genomes used is given in Table 2. In the table, column ‘cov.’ contains the fraction of each chromosome covered by the contigs selected. Column ‘tech.’ describes de sequencing technology used, when this information was available in the corresponding GenBank file. Four of the genomes in Table 2 have two chromosomes; each chromosome was dealt with separately, thus creating a total of 23 test cases. The genomes chosen were those available from GenBank^b^ on December 7, 2011, for which complete genomes were also available (with at least 4 contigs for each genome). These genomes are quite diverse phylogenetically, representing seven different bacterial groups (where “group” comes from the GenBank table of completed genomes and generally corresponds to taxonomic phylum or class) and one archaeal group. No other filter was applied in this selection. The sequencing technologies represented in this table include at least one representative of a genome sequenced with 454, Solexa/Illumina, and/or ABI SOLiD. The genome sequences we used in our tests (draft and complete) are available at the website http://marte.ic.unicamp.br:8747.

**Table 2:** Chromosome Sequences Used in Tests


**Genome**	**Chromosome**	**Size (bp)**	**Contigs**	**cov.(%)**	**tech.**
*Aciduliprofundum boonei* T469	NC_013926	1486778	29	89.36	-
*Bacillus subtilis* 168	NC_000964	4215606	5	99.98	Solexa
*Bifidobacterium longum* DJO10A	NC_010816	2375792	43	60.02	-
*Brucella melitensis* bv 1 16M	NC_003317	2117144	41	91.06	454
*Brucella melitensis* bv 1 16M	NC_003318	1177787	12	99.94	454
*Brucella pinnipedialis* B2 94	NC_015857	2138342	55	87.68	454
*Brucella pinnipedialis* B2 94	NC_015858	1260926	34	84.58	454
*Burkholderia thailandensis* E264	NC_007650	2914771	15	85.33	-
*Burkholderia thailandensis* E264	NC_007651	3809201	26	75.90	-
*Chlamydia muridarum* Nigg	NC_002620	1072950	4	99.09	454
*Clostridium cellulovorans* 743B	NC_014393	5262222	293	94.16	454/Illumina
*Corynebacterium aurimucosum* ATCC 700975	NC_012590	2790189	88	85.71	SOLiD
*Corynebacterium efficiens* YS 314	NC_004369	3147090	118	95.90	454
*Micrococcus luteus* NCTC 2665	NC_012803	2501097	121	76.66	-
*Mycobacterium tuberculosis* H37Ra	NC_009525	4419977	220	83.75	-
*Mycoplasma genitalium* G37	NC_000908	580076	24	78.59	454
*Saccharopolyspora erythraea* NRRL 2338	NC_009142	8212805	237	96.43	454
*Selenomonas sputigena* ATCC 35185	NC_015437	2568361	49	97.16	454
*Stigmatella aurantiaca* DW4 3 1	NC_014623	10260756	466	97.62	-
*Streptococcus pneumoniae* TIGR4	NC_003028	2160842	211	90.98	454
*Vibrio* Ex25	NC_013456	3259580	176	91.77	-
*Vibrio* Ex25	NC_013457	1829445	33	95.31	-
*Yersinia pestis* Nepal516	NC_008149	4534590	17	84.21	SOLiD

For each of the query chromosomes we obtained a list of the 20 closest genomes (excluding the query genome itself), among 1331 complete prokaryotic genomes available at GenBank on May 5, 2011. We used NUCMi [22] (a variation of MUMi [23]) to compute the distance between each of the 1331 genomes and the query genome. Our rationale for selecting 20 closest other genomes instead of only the closest was as follows. In practice, the choice of a reference genome should be guided by phylogenetic distance. The closest genome to the draft genome is the one most likely to yield best results. On the other hand, given the extremely small sample of the prokaryotic world that has been sequenced to date, it is perfectly possible that new strains will be sequenced in the future for which the closest reference genome will not be particularly close. Therefore it is important to understand how the performance of a scaffolding program changes with increased distance between the query and possible reference genomes.

## Results and discussion

We ran each scaffold program on each of the 23 query chromosomes using as reference each of the 20 closest chromosomes to the query chromosome. This resulted in 460 scaffold sets for each program. For a given program and a given set of reference chromosomes, we computed average results over the 23 query chromosomes.

Table 3 shows average results for three cases: when the reference chromosome used was the closest available, then the average (mean) over the 10 closest chromosomes, then the average (mean) over all 20 closest chromosomes. Performance of each program on each test set given by each column in the table. The numbers are percentages of correct adjacencies, averaged over all instances in each column. In bold are the best results. The table is sorted from best to worst in the first column. In all cases the best performance was that of sis (promer). If the median is used rather than the mean, then sis (nucmer) is the best program for the closest case, Mauve is the best for the 10-closest case, and sis (promer) is the best for the 20-closest case (see Additional file 1).

**Table 3:** Average Performance (Correct Adjacencies)


**Programs**	**Closest**	**Top 10**	**Top 20**
sis (promer)	**60.96**	**50.01**	**37.27**
r2cat	59.72	44.19	30.30
Mauve	58.65	46.31	32.17
sis (nucmer)	56.39	43.95	28.55
fillScaffolds (nucmer)	48.47	34.10	21.32
OSLay	46.83	34.03	21.28
fillScaffolds (promer)	44.19	32.94	21.63
CONTIGuator	42.02	30.35	20.01
Projector 2	36.89	25.35	15.51
ABACAS	33.80	23.95	13.20

**Figure 8:**
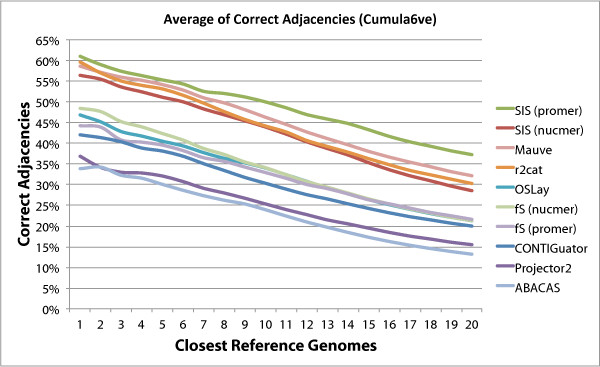
**Average of Correct Adjacencies.** Variation in number of correct adjacencies for each program as the reference genome changes from the closest to the most distant, in a total of 20 reference genomes. fs is fillScaffolds.

**Figure 9:**
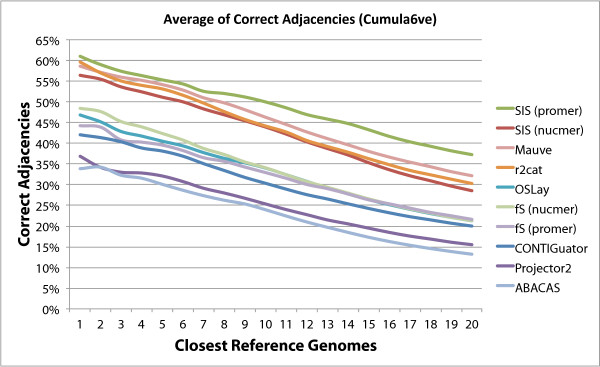
**Average of Correct Adjacencies (Cumulative Version).** Variation in number of correct adjacencies for each program as the reference genome changes from the closest to the most distant, in a total of 20 reference genomes. This is a cumulative version of Figure 9. The points plotted correspond to the cumulative averages as more distant genomes are included. fs is fillScaffolds.

In order to evaluate the performance of the programs tested with increased distance from the reference chromosome we created the graphs shown in Figures 8 and 9. These graphs show that the performance of all programs degrade with increased distance, but again here sis (promer) stays above the others in all cases

The results presented so far are averages. In Table 4 we present results concerning the fraction of the instances in which each program was the winner (provider of the best scaffold). In the table columns do not add up to 100% because of ties. Best results are shown in bold. The table is sorted from best to worst in the first column. Using this measure sis (promer) superiority over the other programs is even more pronounced.

**Table 4:** Best Scaffolds (Correct Adjacencies)


**Programs**	**Closest**	**Top 10**	**Top 20**
sis (promer)	** 56.52**	**59.13**	**64.57**
sis (nucmer)	39.13	27.83	20.87
Mauve	26.09	25.65	23.26
OSLay	21.74	15.65	11.30
fillScaffolds (nucmer)	21.74	11.30	8.70
ABACAS	17.39	11.74	7.61
r2cat	13.04	16.09	15.65
CONTIGuator	13.04	9.57	6.30
Projector 2	8.70	6.09	4.57
fillScaffolds (promer)	0.00	6.96	8.04

Tables 5 and 6 are analogous to Tables 3 and 4, respectively, but show the results in terms of coverage (see Testing Methodology). Also under this measure SIS (promer) was the best program.

**Table 5:** Average Performance (Coverage)


**Programs**	**Closest**	**Top 10**	**Top 20**
sis (promer)	**58.6**	**46.0**	**34.6**
r2cat	56.8	40.4	27.5
Mauve	55.9	41.9	29.5
sis (nucmer)	52.4	39.5	25.6
fillScaffolds (nucmer)	42.9	29.9	19.0
OSLay	42.5	29.0	18.2
CONTIGuator	42.0	29.3	19.3
fillScaffolds (promer)	40.2	29.2	19.5
Projector 2	34.3	22.5	13.9
ABACAS	27.8	19.9	11.3

**Table 6:** Best Scaffolds (Coverage)


**Programs**	**Closest**	**Top 10**	**Top 20**
sis (promer)	**47.8**	**51.7**	**56.5**
sis (nucmer)	39.1	24.4	17.0
Mauve	34.8	25.7	20.2
OSLay	17.4	12.2	8.9
fillScaffolds (nucmer)	17.4	11.3	7.4
r2cat	13.0	17.4	14.4
ABACAS	13.0	11.7	7.8
CONTIGuator	13.0	10.0	6.5
Projector 2	8.7	6.1	4.6
fillScaffolds (promer)	0.0	7.0	7.4

It is important to note that the pairs of query-reference genomes tested were not selected for containing inversions. So any given pair query-reference could have any kind of rearrangement. The success of sis in the cases tested suggests that inversions are indeed widespread in prokaryotic genomes (to the extent that genomes in Table 2 are a representative sample).

### Additional results

Here we briefly describe additional results for which tables and graphs can be found in the Additional file 1.

Another way of parsing results is to ask the question: which program yields best results for each query chromosome sequence, in terms of number of average correct adjacencies? sis (promer and nucmer) are the best programs under this measure.

Another question to ask is what kind of influence does the number of contigs has on a program’s performance? We have found that all programs obtain their best results for chromosome sequences that have few contigs (between 4 and 25). As for worst results, the outcome was less clear, since it is not always the case that the largest number of contigs results in poorest performance. We also determined that fillScaffolds and r2cat seem to be less sensitive to variation in number of contigs in the input than other programs. These results need to be taken with caution, because the bins we used had a relatively small number of instances in each (between five and seven).

Finally, we investigated the influence of duplications on the performance of SIS in terms of correct adjacencies. We found that on average no more than 13% of incorrect adjacencies are due to duplications.

### Running times

Running times of the tests are dominated by the sequence comparison step. For example, in the case of sis, nucmer takes on average 1 minute for a genome pair, and promer takes on average 5 minutes. sis itself takes less than a second to compute a scaffold set. OSLay takes about 3 seconds. After obtaining the conserved blocks, fillScaffolds takes about 3 seconds. r2cat takes between 15 and 20 seconds to determine conserved blocks and about 2 seconds to generate a scaffold. ABACAS takes between 10 and 30 seconds total time. CONTIGuator takes about 2 minutes to determine conserved blocks using BLAST+ and to generate a scaffold. Mauve Aligner takes between 8 and 322 minutes per genome pair (on average, 46 minutes). All these tests were executed on the same machine, a standard desktop computer with Intel Core2 Duo processor, 3.0 GHz and 4 GB RAM. Projector 2 can only be run on a web server. We observed that the total computation time on the server was about 1 minute, when the server appeared to have a light load.

### Details of these tests and other tests

In Additional file 1 we present more detailed results of the tests above as well as results using pairs of real genomes and simulated contigs. sis also came out as the best program in these other tests. These results are a refinement of preliminary tests presented in [24].

#### Availability of SIS

SIS is available as a web server at http://marte.ic.unicamp.br:8747. It can also be freely downloaded as a stand alone program from the same website. SIS generates its scaffolds both as ordered lists of contigs as well as in nucleotide sequence FASTA format.

## Conclusions

We have presented a new linear-time algorithm for generating scaffolds for draft genomes, based on the concept of inversion signatures. We implemented this algorithm creating program sis, which is to our knowledge the first scaffold program that explicitly models the biological phenomenon of replicon inversion in prokaryotes. We compared sis to seven other programs, and demostrated that sis achieves better performance relative to these other programs using a real and diverse suite of test cases.

In a real world setting, the scaffolds generated by sis can help in the gap closure process. For example, sis output can easily be used as input to primer generation programs such as Primer3 [25]. If paired-end read data is available, it can be used to check the scaffolds provided and to connect separate scaffolds, thus generating more complete and reliable contig orderings. Ideally, sis should be able to use paired-end read information, but this will require a substantial change to the algorithm presented here, as evidenced by the sophistication of the recent algorithm for paired-end read scaffolding presented by Gao et al. [1]. Another possible improvement in the scaffold generating process is to use several reference genomes instead of only one, under the rationale that some inversions may be missed by using reference genome *X* but may be detected using some other reference genome *Y *. An idea similar to this is described for the program PGA4genomics [11].

## Endnotes

^a^http://www.ncbi.nlm.nih.gov/genomes/lproks.cgi^b^ftp://ftp.ncbi.nih.gov/genomes/Bacteria∖_DRAFT

## Competing interests

The authors declare that they have no competing interests.

## Authors’ contributions

JCS had the idea of using inversion signatures and sketched the algorithm on which SIS is based. ZD and UD designed the algorithm and implemented SIS. JCS, ZD, and UD designed the tests. ZD and UD carried out the tests. UD created the webserver version. All authors contributed to the writing of the manuscript.

## Supplementary Material

Additional file 1 **Figure S1.** Variation in the Number of Correct Adjacencies (Top 1). Variation in the number of correct adjacencies determined by each scaffold program when the reference genome is the closest to the query genome. The diamond is the median. **Figure S2.** Variation in the Number of Correct Adjacencies (Top 10). Variation in the number of correct adjacencies determined by each scaffold program averaged over the 10 closest genomes to the query genome. The diamond is the median. **Figure S3.** Variation in the Number of Correct Adjacencies (Top 20). Variation in the number of correct adjacencies determined by each scaffold program averaged over the 20 closest genomes to the query genome. The diamond is the median. **Figure S4.** Test Cases X Correct Adjacencies (Top 1). **Figure S5.** Test Cases X Correct Adjacencies (Top 10). **Figure S6.** Test Cases X Correct Adjacencies (Top 20). **Figure S7.** Correct Adjacencies X Number of Contigs (Top 1). **Figure S8.** Correct Adjacencies X Number of Contigs (Top 10). **Figure S9.** Correct Adjacencies X Number of Contigs (Top 20). **Figure S10.** Example of Dotplot. Pairwise whole genome comparison of two *Pseudomonas* species. The comparison was done using nucmer [18]. **Figure S11.***Mycobacterium* (All pairs). Variation of the distribution of the number of correct adjacencies in the scaffolds generated by the various programs for the complete set (210 pairs) of *Mycobacterium* genomes. **Figure S12.***Pseudomonas* (All Pairs). Variation of the distribution of the number of correct adjacencies in the scaffolds generated by the various programs for the complete set (153 pairs) of *Pseudomonadaceae* genomes. **Figure S13.***Shewanellas* (All Pairs). Variation of the distribution of the number of correct adjacencies in the scaffolds generated by the various programs for the complete set (190 pairs) of *Shewanella* genomes. **Figure S14.***Xanthomonas* (All Pairs). Variation of the distribution of the number of correct adjacencies in the scaffolds generated by the various programs for the complete set (36 pairs) of *Xanthomonas* genomes. **Figure S15.***Mycobacterium* (Best Pairs). Variation of the distribution of the number of correct adjacencies in the scaffolds generated by the various programs for only those pairs of *Mycobacterium* genomes that are closest to each other in the second batch of tests. **Figure S16.***Pseudomonas* (Best Pairs). Variation of the distribution of the number of correct adjacencies in the scaffolds generated by the various programs for only those pairs of *Pseudomonas* genomes that are closest to each other in the second batch of tests. **Figure S17.***Shewanellas* (Best Pairs). Variation of the distribution of the number of correct adjacencies in the scaffolds generated by the various programs for only those pairs of *Shewanella* genomes that are closest to each other in the second batch of tests. **Figure S18.***Xanthomonas* (Best Pairs). Variation of the distribution of the number of correct adjacencies in the scaffolds generated by the various programs for only those pairs of *Xanthomonas* genomes that are closest to each other in the second batch of tests. **Figure S19.** Average (All Pairs). Variation of the distribution of the average number of correct adjacencies in the scaffolds generated by the various programs for the complete set of test instances in the second batch. **Figure S20.** Average (Best Pairs). Variation of the distribution of the average number of correct adjacencies in the scaffolds generated by the various programs for only those pairs of genomes that are closest to each other in the second batch of tests. Click here for file
