# Supplementary material for: SIS: a program to generate draft genome sequence scaffolds for prokaryotes
Source: BMC Bioinformatics. 2012 May 14;13:96. doi: 10.1186/1471-2105-13-96 (PMC3674793; doi:10.1186/1471-2105-13-96)
Supplement: Additional file 1 — Figure S1. Variation in the Number of Correct Adjacencies (Top 1). Variation in the number of correct adjacencies determined by each scaffold program when the reference genome is the closest to the query genome. The diamond is the median. Figure S2. Variation in the Number of Correct Adjacencies (Top 10). Variation in the number of correct adjacencies determined by each scaffold program averaged over the 10 closest genomes to the query genome. The diamond is the median. Figure S3. Variation in the Number of Correct Adjacencies (Top 20). Variation in the number of correct adjacencies determined by each scaffold program averaged over the 20 closest genomes to the query genome. The diamond is the median. Figure S4. Test Cases X Correct Adjacencies (Top 1). Figure S5. Test Cases X Correct Adjacencies (Top 10). Figure S6. Test Cases X Correct Adjacencies (Top 20). Figure S7. Correct Adjacencies X Number of Contigs (Top 1). Figure S8. Correct Adjacencies X Number of Contigs (Top 10). Figure S9. Correct Adjacencies X Number of Contigs (Top 20). Figure S10. Example of Dotplot. Pairwise whole genome comparison of two Pseudomonas species. The comparison was done using nucmer [18]. Figure S11.Mycobacterium (All pairs). Variation of the distribution of the number of correct adjacencies in the scaffolds generated by the various programs for the complete set (210 pairs) of Mycobacterium genomes. Figure S12.Pseudomonas (All Pairs). Variation of the distribution of the number of correct adjacencies in the scaffolds generated by the various programs for the complete set (153 pairs) of Pseudomonadaceae genomes. Figure S13.Shewanellas (All Pairs). Variation of the distribution of the number of correct adjacencies in the scaffolds generated by the various programs for the complete set (190 pairs) of Shewanella genomes. Figure S14.Xanthomonas (All Pairs). Variation of the distribution of the number of correct adjacencies in the scaffolds generated by the various programs for the complet [file 1471-2105-13-96-S1.pdf]

SIS: a Program to Generate Draft Genome Sequence Scaffolds for Prokaryotes
Supplementary Materials

Zanoni Dias [1], Ulisses Dias [1] and João C. Setubal[2]

[1]Instituto de Computação, Universidade Estadual de Campinas, Campinas, SP, Brazil
[2]Departamento de Bioquímica, Instituto de Química, Universidade de São Paulo, SP, Brazil

Email: Zanoni Dias - zanoni@ic.unicamp.br; Ulisses Dias - udias@ic.unicamp.br; João C. Setubal*- setubal@iq.usp.br;

*Corresponding author

Boxplots for the tests using real contigs

Here we present boxplots for the tests described in the main text (using real contigs). These boxplots give an idea of the spread of results for a given program and across the genomes tested.
Figure 1 shows the variation in the number of correct adjacencies determined by each scaffold program when the reference genome is the closest to the query genome. Figures 2 and 3 show the boxplots when the correct number of adjcencies is averaged over the results obtained using the 10 closest and 20 closest genomes, respectively. The decrease in performance for all programs when more distant genomes are included is clear. SIS (nucmer) has the best median value in the closest reference genome case; Mauve has the best median value in the 10-closest case, and SIS (promer) has the best median value in the 20-closest case.

Tables with results according to genome

Tables 1, 2, and 3 show the results of all programs on each individual replicon sequence tested. Most programs do well on the replicon sequence with the fewest contigs if only the closest reference genome is used (arguably the "easiest" case). Based on the totals of the last rows in these tables SIS (promer or nucmer) are clearly the best programs.

Variation of performance depending on fraction of correct adjacencies

We separated results based on average number of correct adjacencies according to the following bins: 0-20%, 20-40%, 40-60%, 60-80%, and 80-100%. Thus, for example, a program that gets between 40 and 60% correct adjacencies in 80% of the tests would have a bar with length proportional to 80% in the bar corresponding to the 20-40% bin. The results are shown in Figs. 4, 5, and 6.

Considering the bin with more than 80% correct adjacencies, the graphs show that SIS (promer) is the best program in all three scenarios tested (top 1, top 10, and top 20). The same happens for the cumulative bins with more than 40% correct adjacencies.

Variation of performance depending on number of contigs

We parsed results based on number of contigs in the draft genomes. We separated the 23 replicon sequences in four bins, according to the number of contigs each one has: 1-25, 26-50, 51-200, and 201-500. The results are shown in Figs. 7, 8, and 9.

For the case where replicon sequences have 25 or fewer contigs, SIS (promer or nucmer) are the best programs in all three scenarios. For 26 or more contigs, the results show that there are cases when r2cat or Mauve achieve the best results. The graphs also suggest that r2cat and Mauve are less sensitive to the number of contigs in the input.

As noted in the main text, these results require some caution because of the small number of cases in each bin (between five and seven).

Tests using simulated contigs

For this test we used 21 complete genomes of the *Mycobacterium* genus, 18 complete genomes of the *Pseudomonadaceae* family, 20 complete genomes of the *Shewanella* genus, and 9 complete genomes of the *Xanthomonas* genus. All genomes in these groups have one main circular chromosome. Some members have small plasmids; we do not take plasmids into consideration. Pairwise whole genome comparisons of these genomes show a distinctive 'X' pattern, indicating a predominance of symmetric inversions (See Fig. 10 for an example).

We simulated contigs by the following procedure: each genome was divided up into 100 substrings of the same size. Then a section with length 2.5% of the total contig length was removed from each end, with the goal of simulating the fact that draft genomes usually contain less than the complete genome sequence. The resulting sequence corresponds to 95% of the complete genome. Then the 100 contigs were randomly

shuffled, and with 0.5 probability each contig was reverse complemented. The reverse complementation with 0.5 probability simulates the fact that when contigs are generated in the sequencing and assembly processes they can come out in any of the two possible orientations.

Let $R = \{r_1, r_2, \ldots, r_n\}$ be the original set of genomes in a group and $Q = \{q_1, q_2, \ldots, q_n\}$ be the set of simulated draft genomes obtained by the above process, where q_i is the draft genome obtained from r_i. We carried out $n(n-1)$ tests for each program and each group (with values of n being 21, 18, 20, and 9), where in each test r_i is the reference genome and q_j is the draft genome, with $i \neq j$.

The performance comparison was done against the same seven scaffold generating programs mentioned in the main text, namely ABACAS [1], fillScaffolds [2], Mauve Aligner [3], OSLay [4], Projector 2 [5], r2cat [6], and CONTIGuator [7].

Results

Figs. 11, 12, 13 and 14 shows the results averaging over all pairs of genomes for each program and for each bacterial group.

In practice, and as stated in the main text, the choice of a reference genome would be guided by phylogenetic distance. The closest genome to the draft genome is the one most likely to yield best results. With this in mind we computed the pairs of closest *Mycobacterium*, *Pseudomonadaceae*, *Shewanella* and *Xanthomonas* genomes based on the genomic distance MUMi [8], and tabulated the outcomes for these pairs only. The results are shown in Fig. 15, 16, 17 and 18, respectively.

In Figs. 19 and 20 we present boxplots in which the results for all four bacterial groups are combined and averaged. Using the mean values for this combination, SIS (promer and nucmer) come out as the best programs. When all pairs of genomes are used to obtain the mean, SIS (promer) averages 58.4 correct adjacencies and SIS (nucmer) averages and 46.3. Mauve is the third best program with 28.2 correct adjacencies. Averaging only over the best pairs, SIS (promer) obtains 82.6 correct adjcencies and SIS (nucmer) 82.2. The third best program under this measure is again Mauve, with 67.8 correct adjacencies.

Effect of duplications

We investigated the effect of genome duplications on the performance of SIS. For each test case (from the test set of the main text) we determined the existence of duplicated segments of size at least k bp, with k varying from a minimum of 100 to a maximum of 2000. For each value of k we determined the contigs that contained such duplicated segments, and placed them in set D. Duplications were determined by running

nucmer (or promer) on the pair of genomes (draft, reference) and then script `delta-filter` (part of the MUMmer package). With SIS results in hand, we computed correct adjacencies in contigs in D and contigs not in D. Tables 4 and 5 show the results for all pairs for which set D was not empty. The decrease in average performance did not exceed 13 percentage points.

References

1. Assefa S, Keane TM, Otto TD, Newbold C, Berriman M: **ABACAS: algorithm-based automatic contiguation of assembled sequences**. *Bioinformatics* 2009, **25**:1968–1969.
2. Munoz A, Zheng C, Zhu Q, Albert VA, Rounsley S, Sankoff D: **Scaffold filling, contig fusion and comparative gene order inference**. *BMC Bioinformatics* 2010, **11**:304.
3. Rissman AI, Mau B, Biehl BS, Darling AE, Glasner JD, Perna NT: **Reordering contigs of draft genomes using the Mauve aligner**. *Bioinformatics* 2009, **25**:2071–2073.
4. Richter DC, Schuster SC, Huson DH: **OSLay: optimal syntenic layout of unfinished assemblies**. *Bioinformatics* 2007, **23**:1573–1579.
5. van Hijum SA, Zomer AL, Kuipers OP, Kok J: **Projector 2: contig mapping for efficient gap-closure of prokaryotic genome sequence assemblies**. *Nucleic Acids Res.* 2005, **33**:W560–566.
6. Husemann P, Stoye J: **r2cat: synteny plots and comparative assembly**. *Bioinformatics* 2010, **26**:570–571.
7. Galardini M, Biondi EG, Bazzicalupo M, Mengoni A: **CONTIGuator: a bacterial genomes finishing tool for structural insights on draft genomes**. *Source Code for Biology and Medicine* 2011, **6**(11).
8. Deloger M, El Karoui M, Petit MA: **A genomic distance based on MUM indicates discontinuity between most bacterial species and genera**. *J. Bacteriol.* 2009, **191**:91–99.
9. Kurtz S, Phillippy A, Delcher AL, Smoot M, Shumway M, Antonescu C, Salzberg SL: **Versatile and open software for comparing large genomes**. *Genome Biol.* 2004, **5**(2):R12.

Figures

Figure 1 - Variation in the Number of Correct Adjacencies (Top 1)

Variation in the number of correct adjacencies determined by each scaffold program when the reference genome is the closest to the query genome. The diamond is the median. File S1.pdf.

Figure 2 - Variation in the Number of Correct Adjacencies (Top 10)

Variation in the number of correct adjacencies determined by each scaffold program averaged over the 10 closest genomes to the query genome. The diamond is the median. File S2.pdf.

Figure 3 - Variation in the Number of Correct Adjacencies (Top 20)

Variation in the number of correct adjacencies determined by each scaffold program averaged over the 20 closest genomes to the query genome. The diamond is the median. File S3.pdf.

Figure 4 - Test Cases X Correct Adjacencies (Top 1)

File S4.pdf.

Figure 5 - Test Cases X Correct Adjacencies (Top 10)

File S5.pdf.

Figure 6 - Test Cases X Correct Adjacencies (Top 20)

File S6.pdf.

Figure 7 - Correct Adjacencies X Number of Contigs (Top 1)

File S7.pdf.

Figure 8 - Correct Adjacencies X Number of Contigs (Top 10)

File S8.pdf.

Figure 9 - Correct Adjacencies X Number of Contigs (Top 20)

File S9.pdf.

Figure 10 - Example of Dotplot

Pairwise whole genome comparison of two *Pseudomonas* species. The comparison was done using `nucmer` [9]. File S10.pdf.

Figure 11 - Mycobacterium (All pairs)

Variation of the distribution of the number of correct adjacencies in the scaffolds generated by the various programs for the complete set (210 pairs) of *Mycobacterium* genomes. File S11.pdf.

Figure 12 - Pseudomonas (All Pairs)

Variation of the distribution of the number of correct adjacencies in the scaffolds generated by the various programs for the complete set (153 pairs) of *Pseudomonadaceae* genomes. File S12.pdf.

Figure 13 - Shewanellas (All Pairs)

Variation of the distribution of the number of correct adjacencies in the scaffolds generated by the various programs for the complete set (190 pairs) of *Shewanella* genomes. File S13.pdf.

Figure 14 - Xanthomonas (All Pairs)

Variation of the distribution of the number of correct adjacencies in the scaffolds generated by the various programs for the complete set (36 pairs) of *Xanthomonas* genomes. File S14.pdf.

Figure 15 - Mycobacterium (Best Pairs)

Variation of the distribution of the number of correct adjacencies in the scaffolds generated by the various programs for only those pairs of *Mycobacterium* genomes that are closest to each other in the second batch of tests. File S15.pdf.

Figure 16 - Pseudomonas (Best Pairs)

Variation of the distribution of the number of correct adjacencies in the scaffolds generated by the various programs for only those pairs of *Pseudomonas* genomes that are closest to each other in the second batch of tests. File S16.pdf.

Figure 17 - Shewanellas (Best Pairs)

Variation of the distribution of the number of correct adjacencies in the scaffolds generated by the various programs for only those pairs of *Shewanella* genomes that are closest to each other in the second batch of tests. File S17.pdf.

Figure 18 - Xanthomonas (Best Pairs)

Variation of the distribution of the number of correct adjacencies in the scaffolds generated by the various programs for only those pairs of *Xanthomonas* genomes that are closest to each other in the second batch of tests. File S18.pdf.

Figure 19 - Average (All Pairs)

Variation of the distribution of the average number of correct adjacencies in the scaffolds generated by the various programs for the complete set of test instances in the second batch. File S19.pdf.

Figure 20 - Average (Best Pairs)

Variation of the distribution of the average number of correct adjacencies in the scaffolds generated by the various programs for only those pairs of genomes that are closest to each other in the second batch of tests. File S20.pdf.

Tables

Table 1 - Program Performance Using Closest Reference Genome

Program performance per replicon sequence, using closest reference genome. The 'X' in each column (program) and row (replicon) means that that program was the one that yielded the best average of correct adjacencies for that replicon sequence. 'X's in the same row indicate ties. 'fs' stands for fillScaffolds.

	Contigs	SIS (nucmer)	SIS (promer)	ABACAS	OSLay	r2cat	Projector2	Mauve	fs (nucmer)	fs (promer)	CONTIGuator
Chlamydia muridarum Nigg	4	X	X		X	X	X	X	X		X
Bacillus subtilis 168	5	X	X		X	X	X				
Brucella melitensis bv 1 16M	12	X									
Burkholderia thailandensis E264	15	X	X		X			X			X
Yersinia pestis Nepal516	17	X									
Mycoplasma genitalium G37	24		X								
Burkholderia thailandensis E264	26	X	X		X			X	X		X
Aciduliprofundum boonei T469	29		X								
Vibrio Ex25	33		X								
Brucella pinnipedialis B2 94	34	X		X	X				X		
Brucella melitensis bv 1 16M	41	X		X					X		
Bifidobacterium longum DJO10A	43					X					
Selenomonas sputigena ATCC 35185	49		X								
Brucella pinnipedialis B2 94	55	X		X					X		
Corynebacterium aurimucosum ATCC 700975	88							X			
Corynebacterium efficiens YS 314	118		X								
Micrococcus luteus NCTC 2665	121		X								
Vibrio Ex25	176							X			
Streptococcus pneumoniae TIGR4	211							X			
Mycobacterium tuberculosis H37Ra	220			X							
Saccharopolyspora erythraea NRRL 2338	237		X								
Clostridium cellulovorans 743B	293		X								
Stigmatella aurantiaca DW4 3 1	466		X								
Total		9	13	4	5	3	2	6	5	0	3

Table 2 - Program Performance Using 10 Closest Reference Genomes

Program performance per replicon sequence, using 10 closest reference genomes. Meaning of 'X' is the same as in Table 1. 'fs' stands for fillScaffolds.

	Contigs	SIS (nucmer)	SIS (promer)	ABACAS	OSLay	r2cat	Projector2	Mauve	fs (nucmer)	fs (promer)	CONTIGuator
Chlamydia muridarum Nigg	4	X									
Bacillus subtilis 168	5		X								
Brucella melitensis bv 1 16M	12	X									
Burkholderia thailandensis E264	15	X									
Yersinia pestis Nepal516	17	X									
Mycoplasma genitalium G37	24									X	
Burkholderia thailandensis E264	26	X									
Aciduliprofundum boonei T469	29		X								
Vibrio Ex25	33		X								
Brucella pinnipedialis B2 94	34				X						
Brucella melitensis bv 1 16M	41	X									
Bifidobacterium longum DJO10A	43							X			
Selenomonas sputigena ATCC 35185	49		X								
Brucella pinnipedialis B2 94	55			X							
Corynebacterium aurimucosum ATCC 700975	88		X								
Corynebacterium efficiens YS 314	118		X								
Micrococcus luteus NCTC 2665	121		X								
Vibrio Ex25	176		X								
Streptococcus pneumoniae TIGR4	211							X			
Mycobacterium tuberculosis H37Ra	220					X					
Saccharopolyspora erythraea NRRL 2338	237		X								
Clostridium cellulovorans 743B	293		X								
Stigmatella aurantiaca DW4 3 1	466		X								
Total		6	11	1	1	1	0	2	0	1	0

Table 3 - Program Performance Using 20 Closest Reference Genomes

Program performance per replicon sequence, using 20 closest reference genomes. Meaning of 'X' is the same as in Table 1. 'fs' stands for fillScaffolds.

	Contigs	SIS (nucmer)	SIS (promer)	ABACAS	OSLay	r2cat	Projector2	Mauve	fs (nucmer)	fs (promer)	CONTIGuator
Chlamydia muridarum Nigg	4		X								
Bacillus subtilis 168	5		X								
Brucella melitensis bv 1 16M	12	X									
Burkholderia thailandensis E264	15	X									
Yersinia pestis Nepal516	17		X								
Mycoplasma genitalium G37	24									X	
Burkholderia thailandensis E264	26	X									
Aciduliprofundum boonei T469	29		X								
Vibrio Ex25	33		X								
Brucella pinnipedialis B2 94	34				X						
Brucella melitensis bv 1 16M	41		X								
Bifidobacterium longum DJO10A	43							X			
Selenomonas sputigena ATCC 35185	49		X								
Brucella pinnipedialis B2 94	55		X								
Corynebacterium aurimucosum ATCC 700975	88		X								
Corynebacterium efficiens YS 314	118		X								
Micrococcus luteus NCTC 2665	121		X								
Vibrio Ex25	176		X								
Streptococcus pneumoniae TIGR4	211							X			
Mycobacterium tuberculosis H37Ra	220		X								
Saccharopolyspora erythraea NRRL 2338	237		X								
Clostridium cellulovorans 743B	293		X								
Stigmatella aurantiaca DW4 3 1	466		X								
Total		3	16	0	1	0	0	2	0	1	0

Table 4 - Effect of Duplication for Contigs in Set D

Effect of duplications on SIS performance. Correct adjacencies (as percentages) for contigs in set D. Top1 uses only the closest genome; Top10 uses the 10 closest genomes; and Top20 uses the 20 closest genomes.

	SIS (nucmer) %				SIS (promer) %			
k	Top1	Top10	Top20	mean	Top1	Top10	Top20	mean
100	58.13	52.54	40.46	50.38	57.98	54.46	42.77	51.74
500	52.83	43.93	36.64	44.47	51.58	44.80	39.04	45.14
1000	49.93	47.75	40.71	46.13	46.25	47.90	42.09	45.41
1500	47.77	47.91	34.61	43.43	41.00	48.18	38.98	42.72
2000	42.66	34.57	24.68	33.97	29.17	39.19	32.31	33.55
mean	50.26	45.34	35.42		45.20	46.91	39.04	

Table 5 - Effect of Duplication for Contigs Not in Set D

Effect of duplications on SIS performance. Correct adjacencies (as percentages) for contigs *not* in set D. Top1 uses only the closest genome; Top10 uses the 10 closest genomes; and Top20 uses the 20 closest genomes.

	SIS (nucmer) %				SIS (promer) %			
k	Top1	Top10	Top20	mean	Top1	Top10	Top20	mean
100	65.11	58.69	47.19	57.00	64.57	59.98	51.85	58.80
500	63.17	51.72	42.67	52.52	61.70	53.95	47.18	54.28
1000	57.77	54.56	46.13	52.82	57.24	59.22	50.35	55.60
1500	51.50	50.84	39.17	47.17	53.07	57.50	46.19	52.25
2000	49.09	42.13	31.72	40.98	52.23	48.28	37.92	46.14
mean	57.33	51.59	41.38		57.76	55.79	46.70	

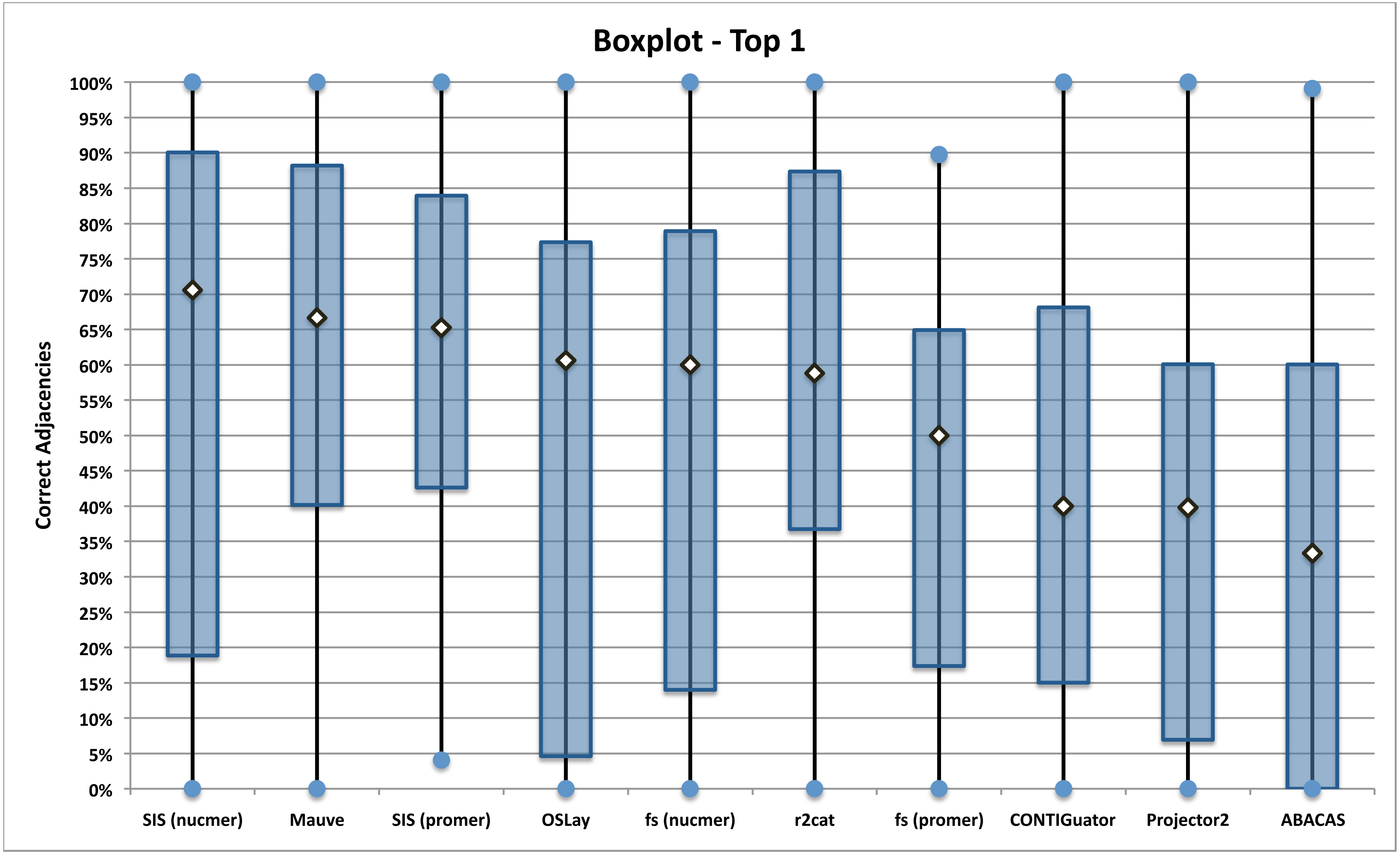
Boxplot - Top 1
Correct Adjacencies
100%
95%
90%
85%
80%
75%
70%
65%
60%
55%
50%
45%
40%
35%
30%
25%
20%
15%
10%
5%
0%
SIS (nucmer)
Mauve
SIS (promer)
OSLay
fs (nucmer)
r2cat
fs (promer)
CONTIGuator
Projector2
ABACAS

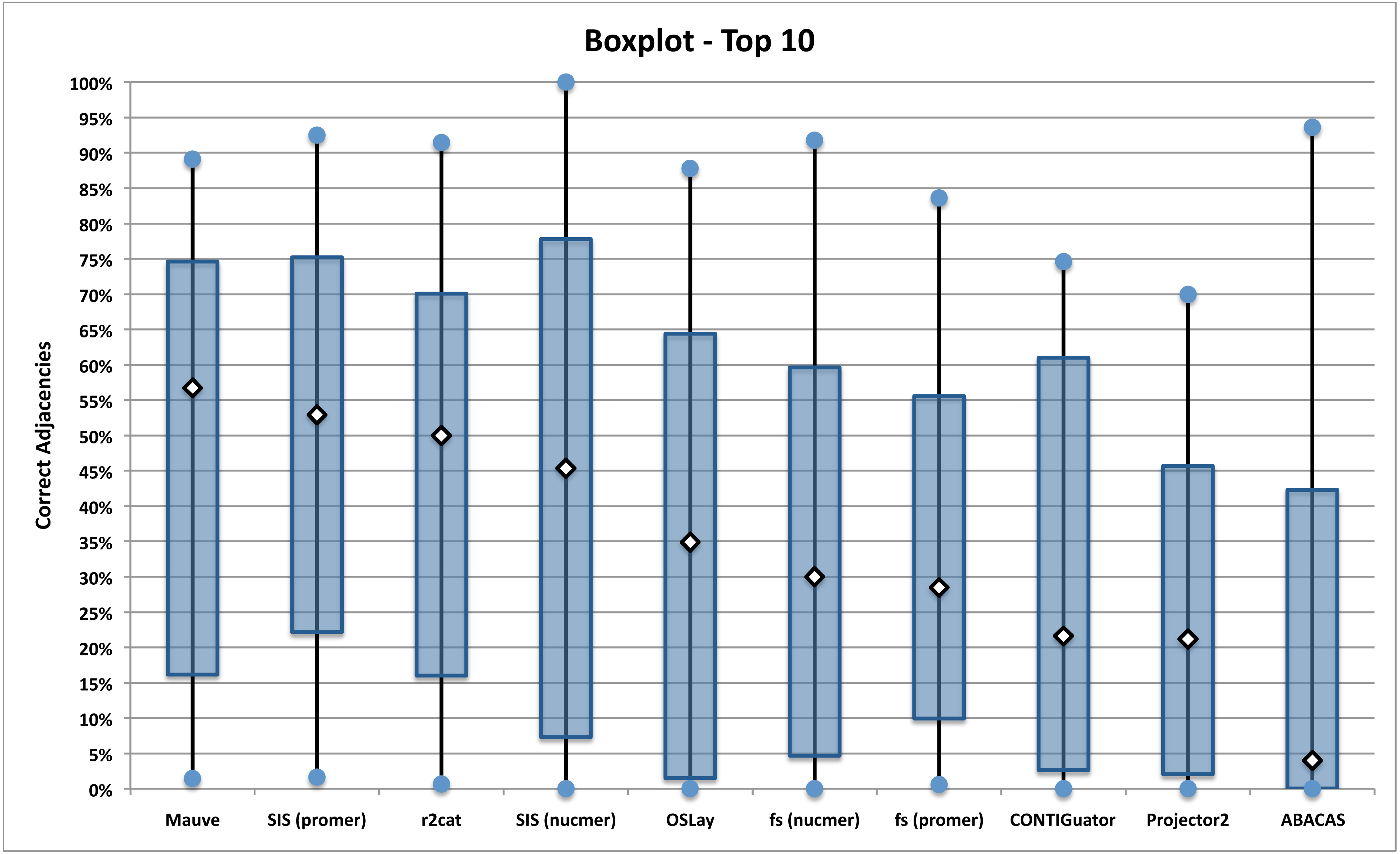
Boxplot - Top 10
Correct Adjacencies
100%
95%
90%
85%
80%
75%
70%
65%
60%
55%
50%
45%
40%
35%
30%
25%
20%
15%
10%
5%
0%
Mauve
SIS (promer)
r2cat
SIS (nucmer)
OSLay
fs (nucmer)
fs (promer)
CONTIGuator
Projector2
ABACAS

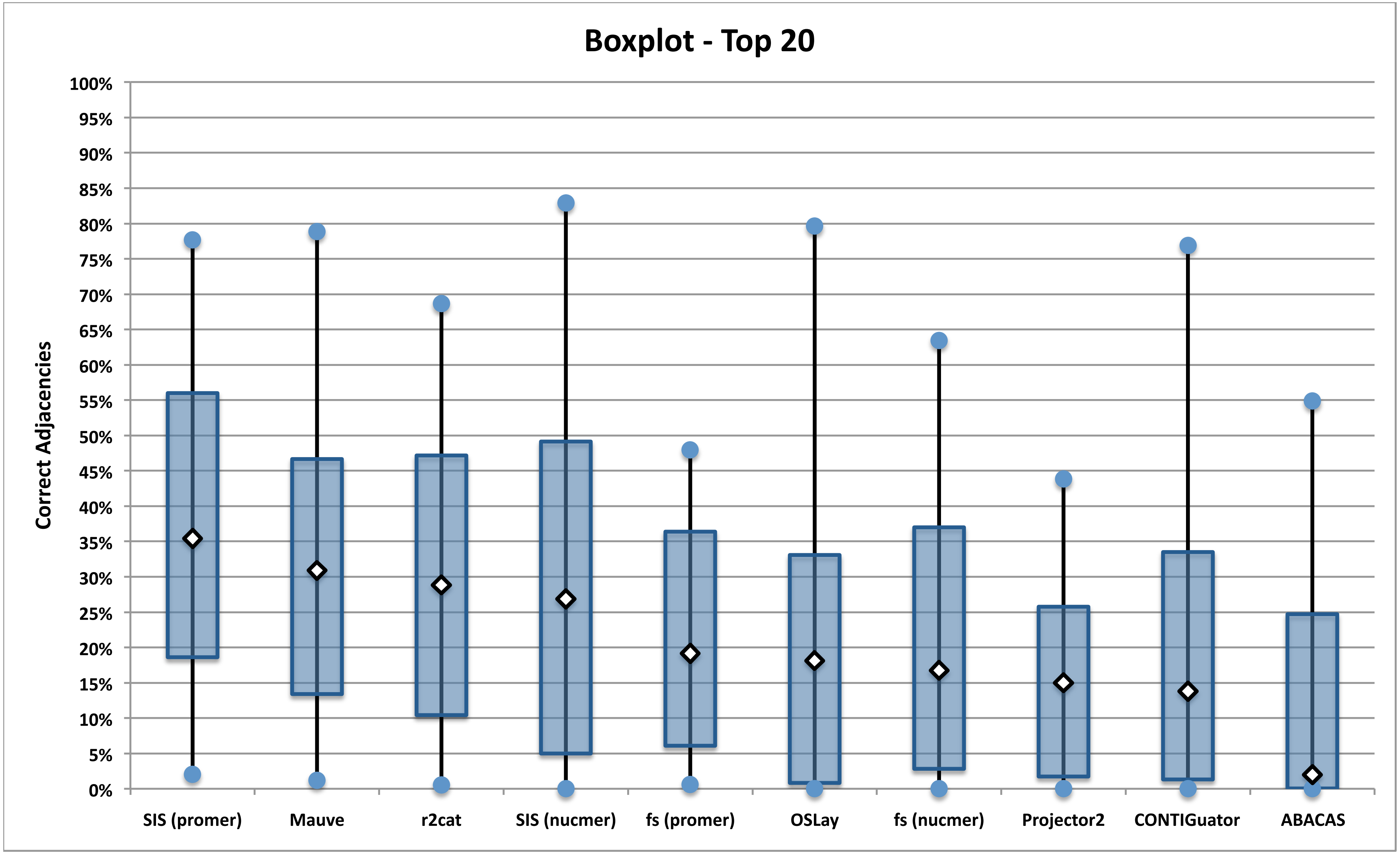
Boxplot - Top 20
Correct Adjacencies
100%
95%
90%
85%
80%
75%
70%
65%
60%
55%
50%
45%
40%
35%
30%
25%
20%
15%
10%
5%
0%
SIS (promer)
Mauve
r2cat
SIS (nucmer)
fs (promer)
OSLay
fs (nucmer)
Projector2
CONTIGuator
ABACAS

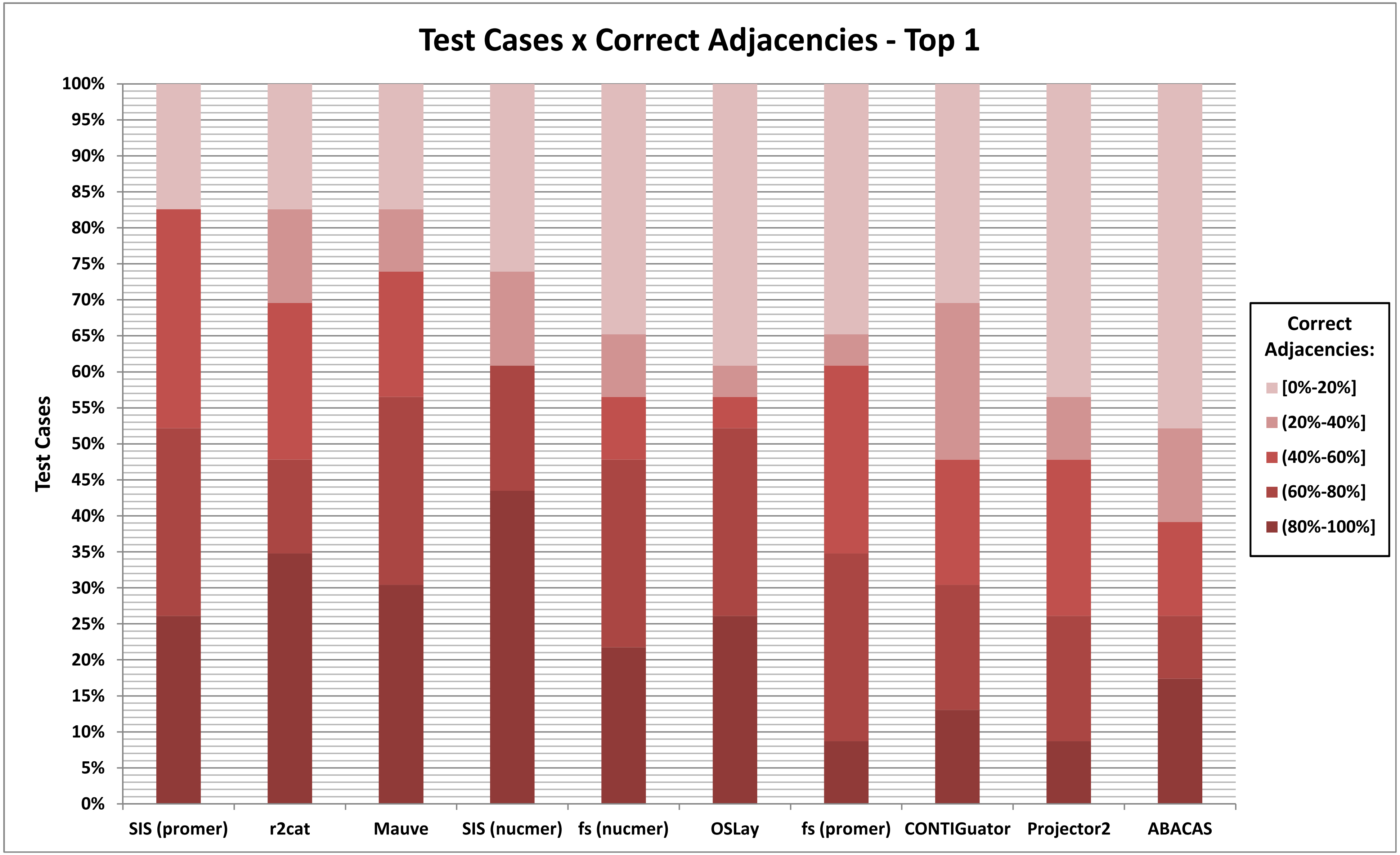
Test Cases x Correct Adjacencies - Top 1
Test Cases
100%
95%
90%
85%
80%
75%
70%
65%
60%
55%
50%
45%
40%
35%
30%
25%
20%
15%
10%
5%
0%
SIS (promer)
r2cat
Mauve
SIS (nucmer)
fs (nucmer)
OSLay
fs (promer)
CONTIGuator
Projector2
ABACAS
Correct Adjacencies:
[0%-20%]
(20%-40%]
(40%-60%]
(60%-80%]
(80%-100%]

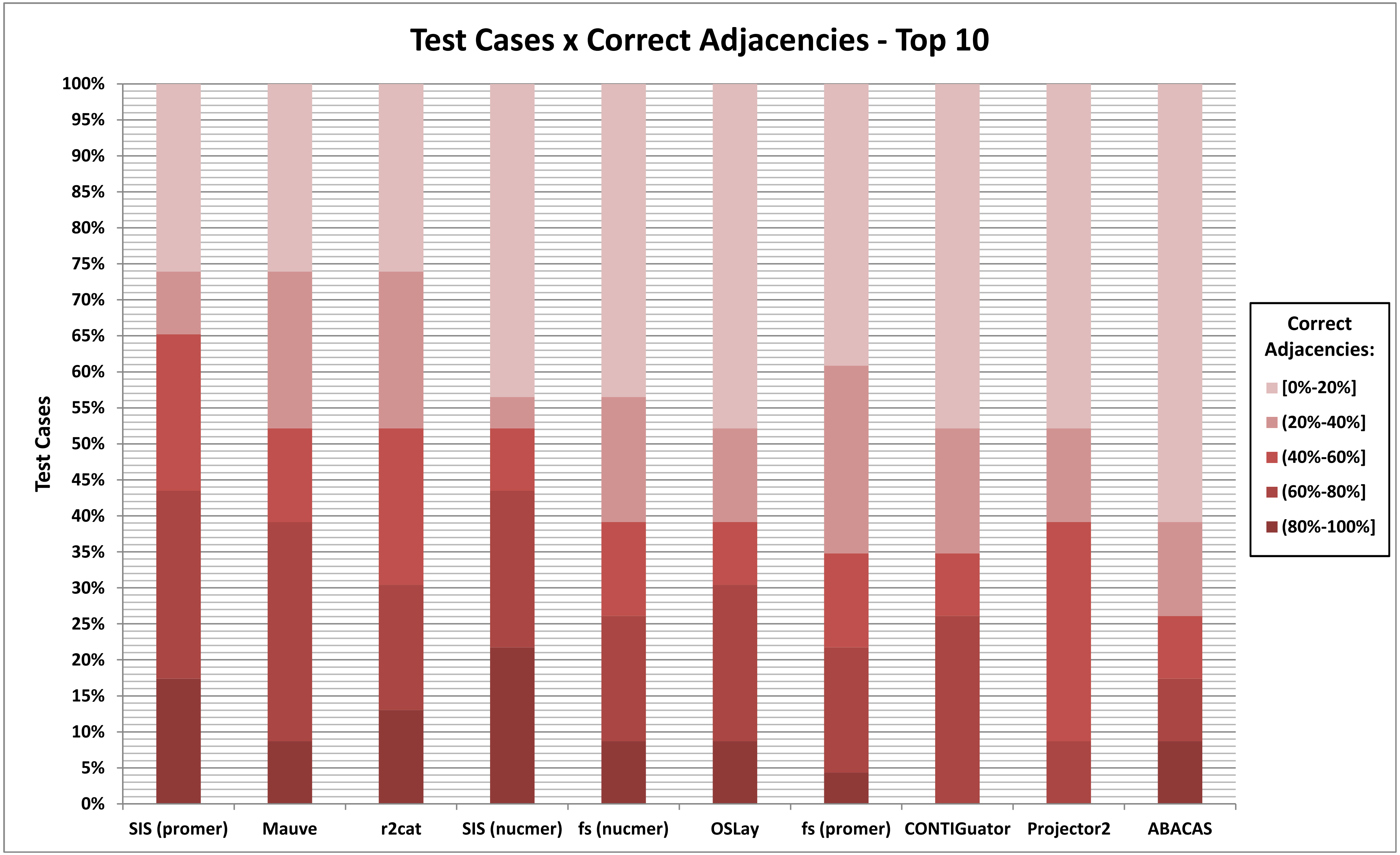
Test Cases x Correct Adjacencies - Top 10
Test Cases
100%
95%
90%
85%
80%
75%
70%
65%
60%
55%
50%
45%
40%
35%
30%
25%
20%
15%
10%
5%
0%
SIS (promer)
Mauve
r2cat
SIS (nucmer)
fs (nucmer)
OSLay
fs (promer)
CONTIGuator
Projector2
ABACAS
Correct Adjacencies:
[0%-20%]
(20%-40%]
(40%-60%]
(60%-80%]
(80%-100%]

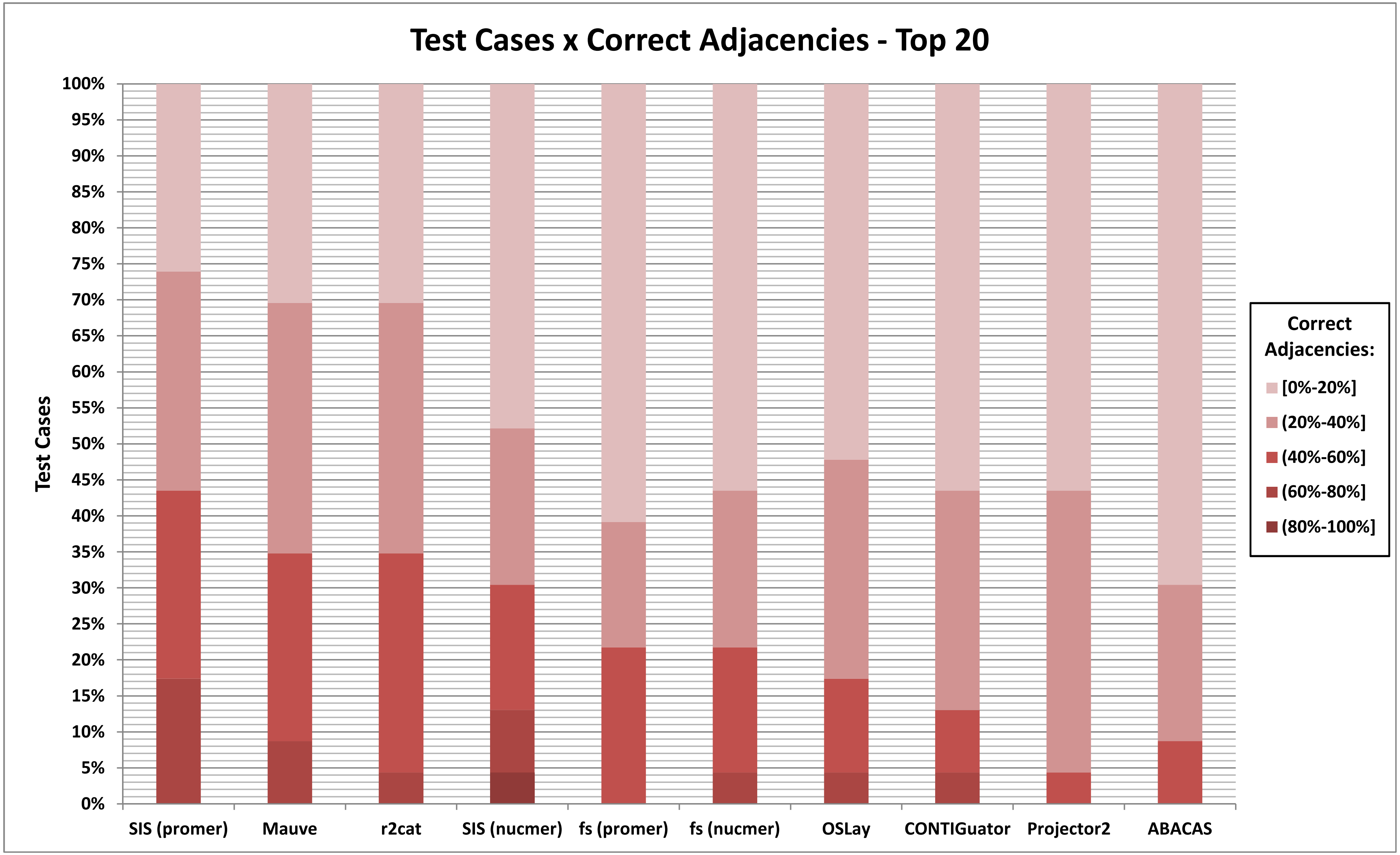
Test Cases x Correct Adjacencies - Top 20
Test Cases
100%
95%
90%
85%
80%
75%
70%
65%
60%
55%
50%
45%
40%
35%
30%
25%
20%
15%
10%
5%
0%
SIS (promer)
Mauve
r2cat
SIS (nucmer)
fs (promer)
fs (nucmer)
OSLay
CONTIGuator
Projector2
ABACAS
Correct Adjacencies:
[0%-20%]
(20%-40%]
(40%-60%]
(60%-80%]
(80%-100%]

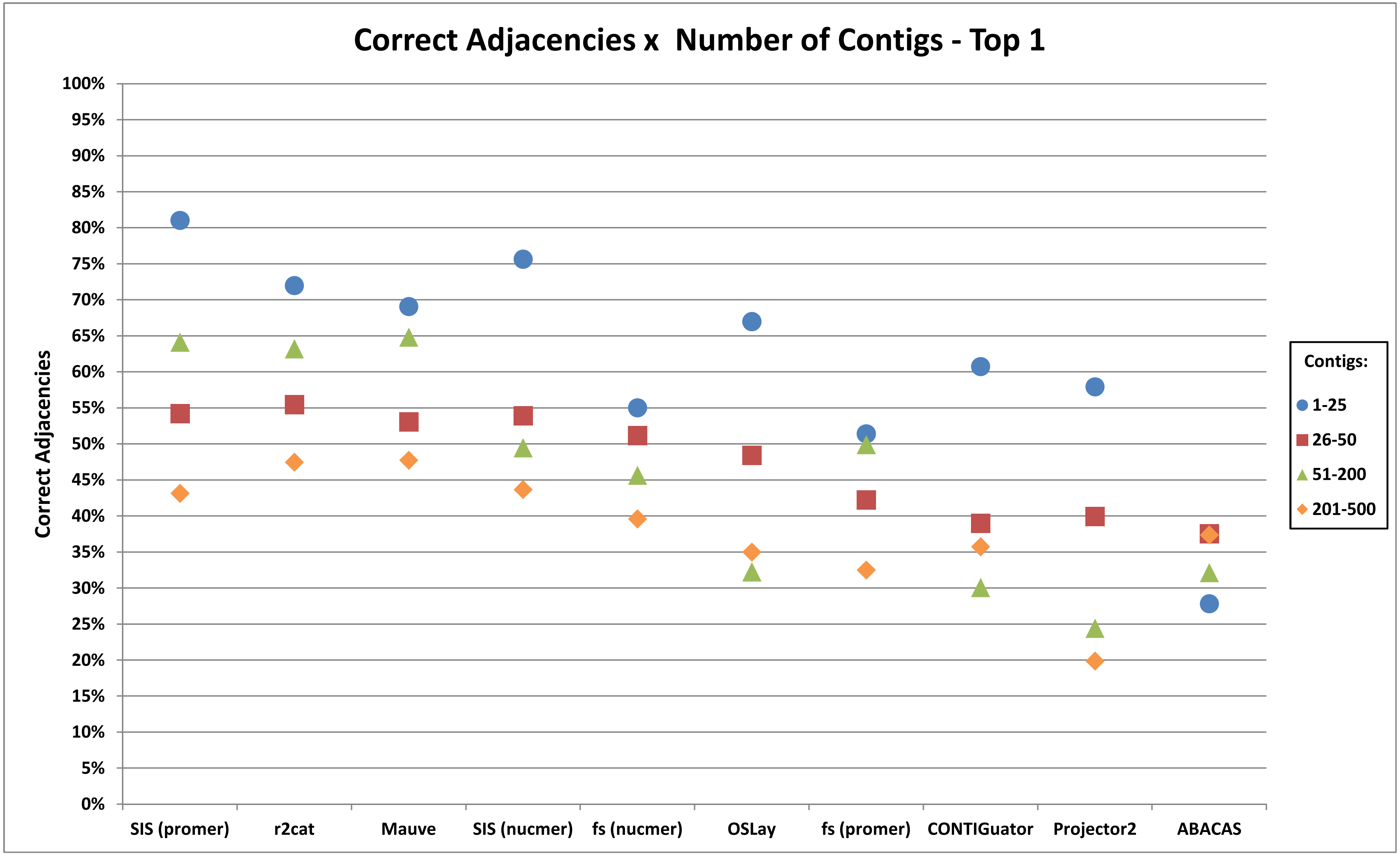
Correct Adjacencies x Number of Contigs - Top 1
Correct Adjacencies
100%
95%
90%
85%
80%
75%
70%
65%
60%
55%
50%
45%
40%
35%
30%
25%
20%
15%
10%
5%
0%
SIS (promer)
r2cat
Mauve
SIS (nucmer)
fs (nucmer)
OSLay
fs (promer)
CONTIGuator
Projector2
ABACAS
Contigs:
1-25
26-50
51-200
201-500

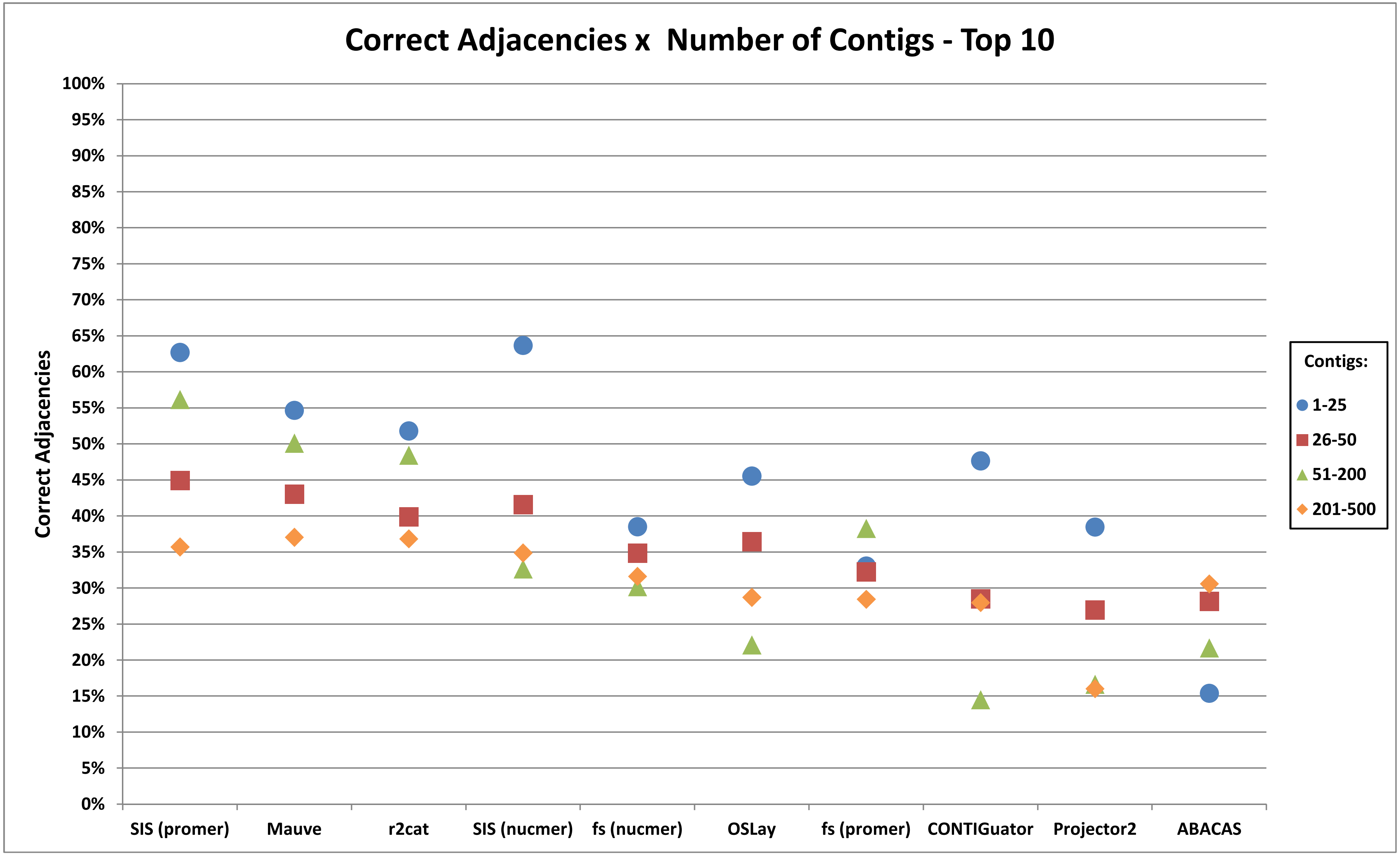
Correct Adjacencies x Number of Contigs - Top 10
Correct Adjacencies
100%
95%
90%
85%
80%
75%
70%
65%
60%
55%
50%
45%
40%
35%
30%
25%
20%
15%
10%
5%
0%
SIS (promer)
Mauve
r2cat
SIS (nucmer)
fs (nucmer)
OSLay
fs (promer)
CONTIGuator
Projector2
ABACAS
Contigs:
1-25
26-50
51-200
201-500

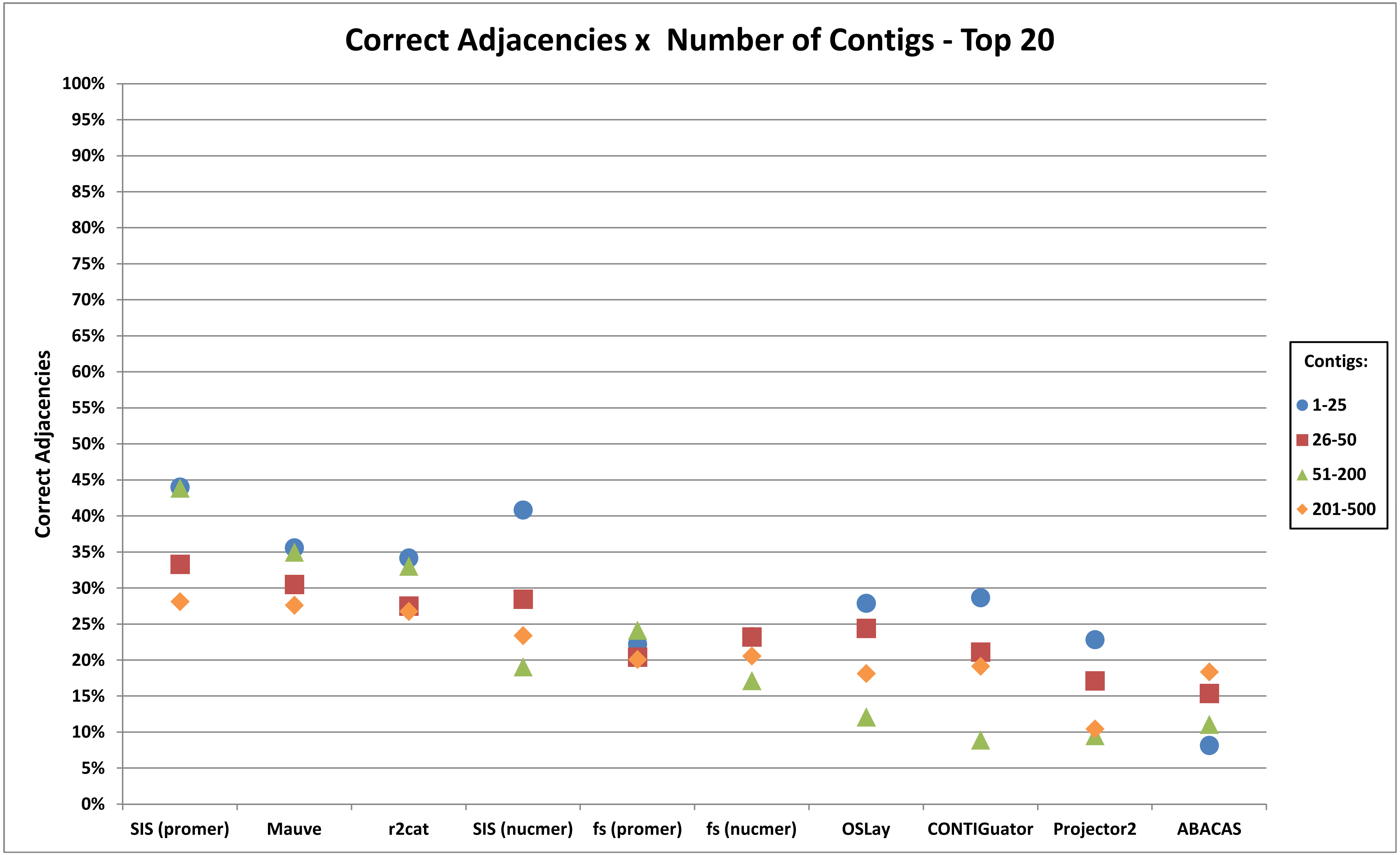
Correct Adjacencies x Number of Contigs - Top 20
Correct Adjacencies
100%
95%
90%
85%
80%
75%
70%
65%
60%
55%
50%
45%
40%
35%
30%
25%
20%
15%
10%
5%
0%
SIS (promer)
Mauve
r2cat
SIS (nucmer)
fs (promer)
fs (nucmer)
OSLay
CONTIGuator
Projector2
ABACAS
Contigs:
1-25
26-50
51-200
201-500

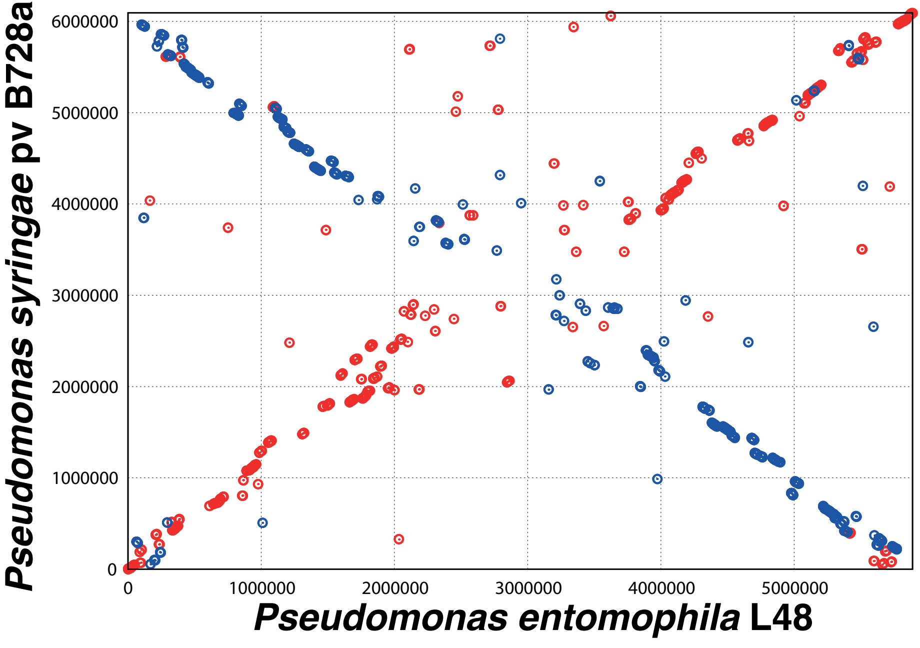

Pseudomonas syringae pv B728a
Pseudomonas entomophila L48
6000000
5000000
4000000
3000000
2000000
1000000
0
0
1000000
2000000
3000000
4000000
5000000

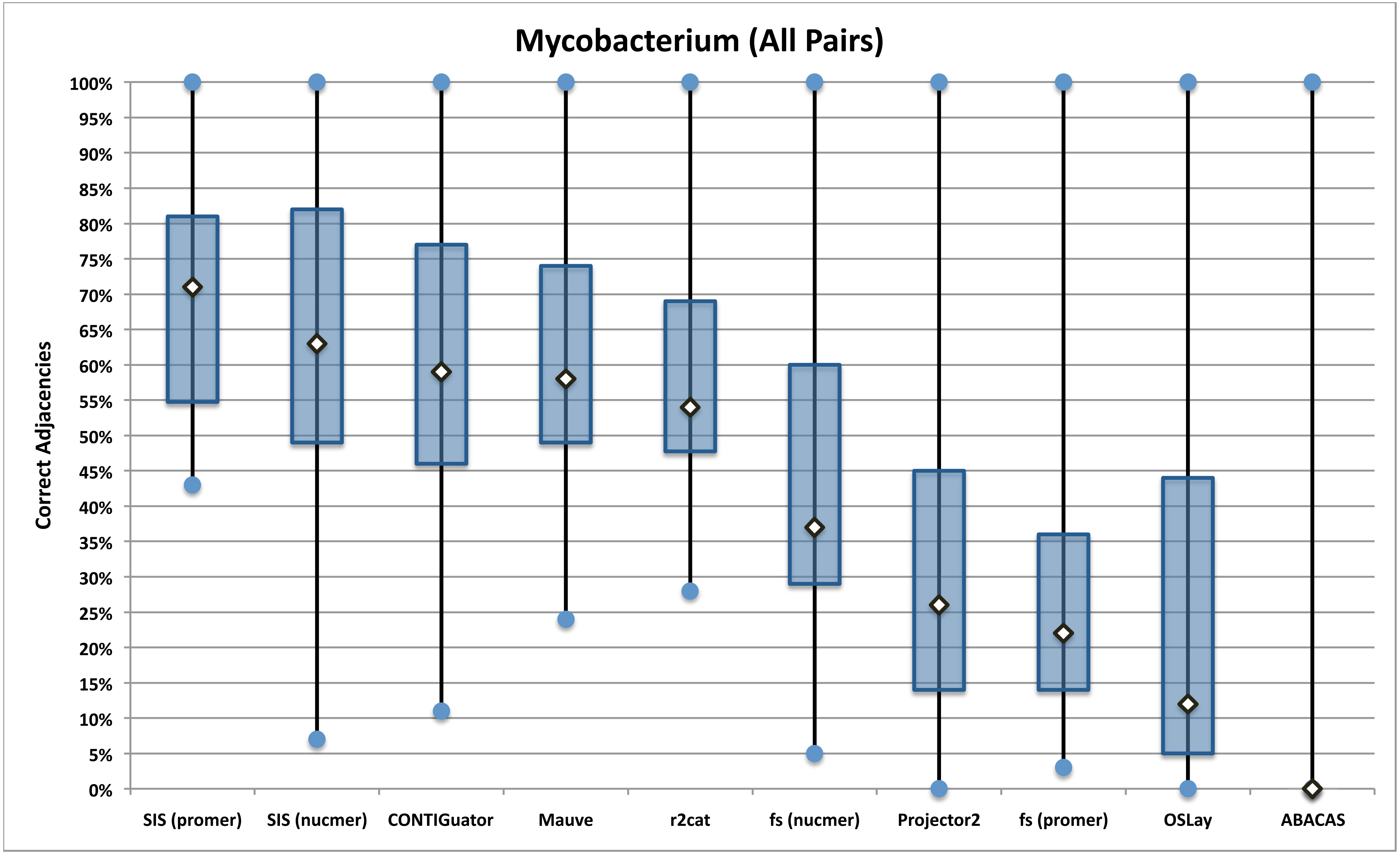
Mycobacterium (All Pairs)
Correct Adjacencies
100%
95%
90%
85%
80%
75%
70%
65%
60%
55%
50%
45%
40%
35%
30%
25%
20%
15%
10%
5%
0%
SIS (promer)
SIS (nucmer)
CONTIGuator
Mauve
r2cat
fs (nucmer)
Projector2
fs (promer)
OSLay
ABACAS

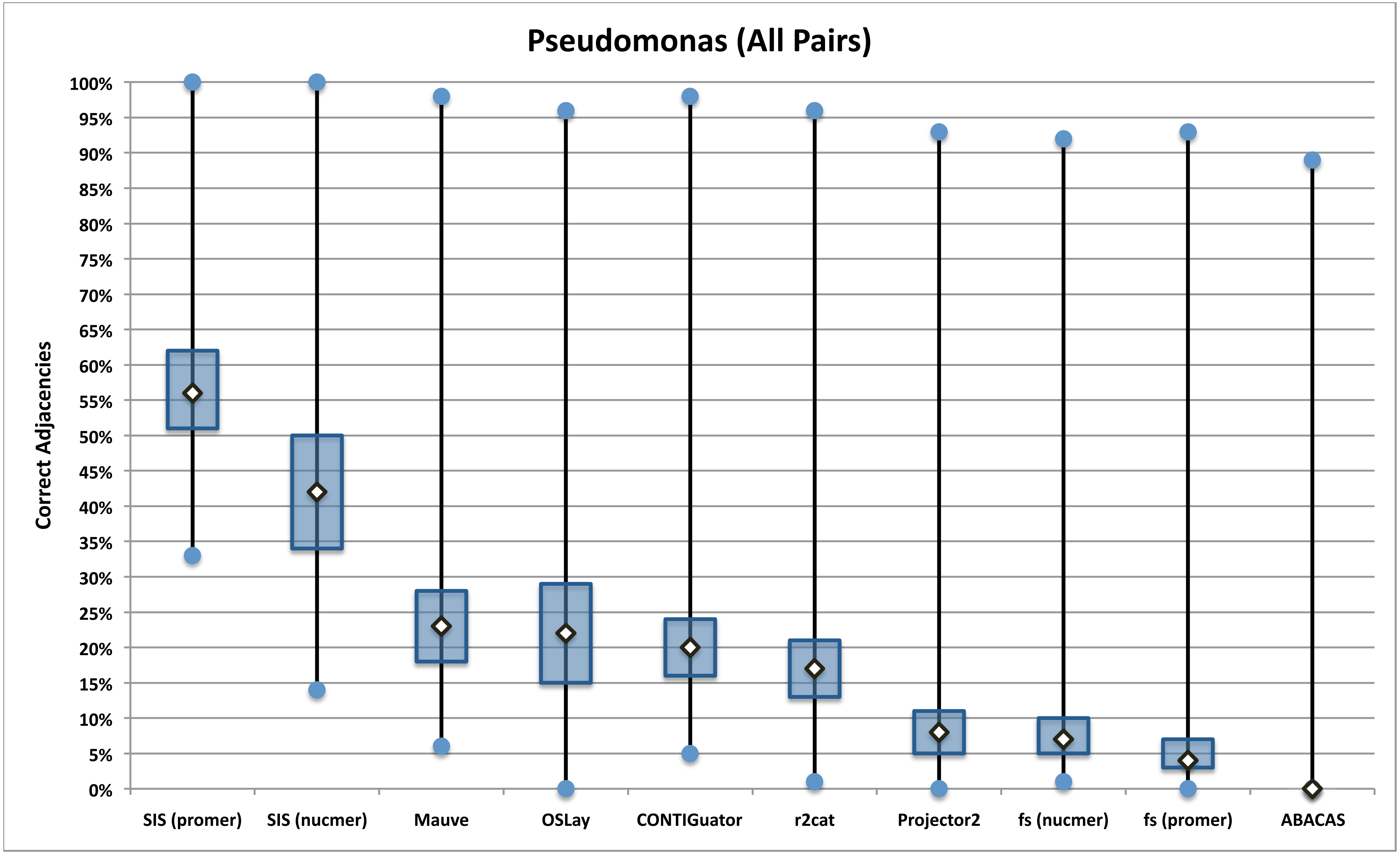
Pseudomonas (All Pairs)
Correct Adjacencies
100%
95%
90%
85%
80%
75%
70%
65%
60%
55%
50%
45%
40%
35%
30%
25%
20%
15%
10%
5%
0%
SIS (promer)
SIS (nucmer)
Mauve
OSLay
CONTIGuator
r2cat
Projector2
fs (nucmer)
fs (promer)
ABACAS

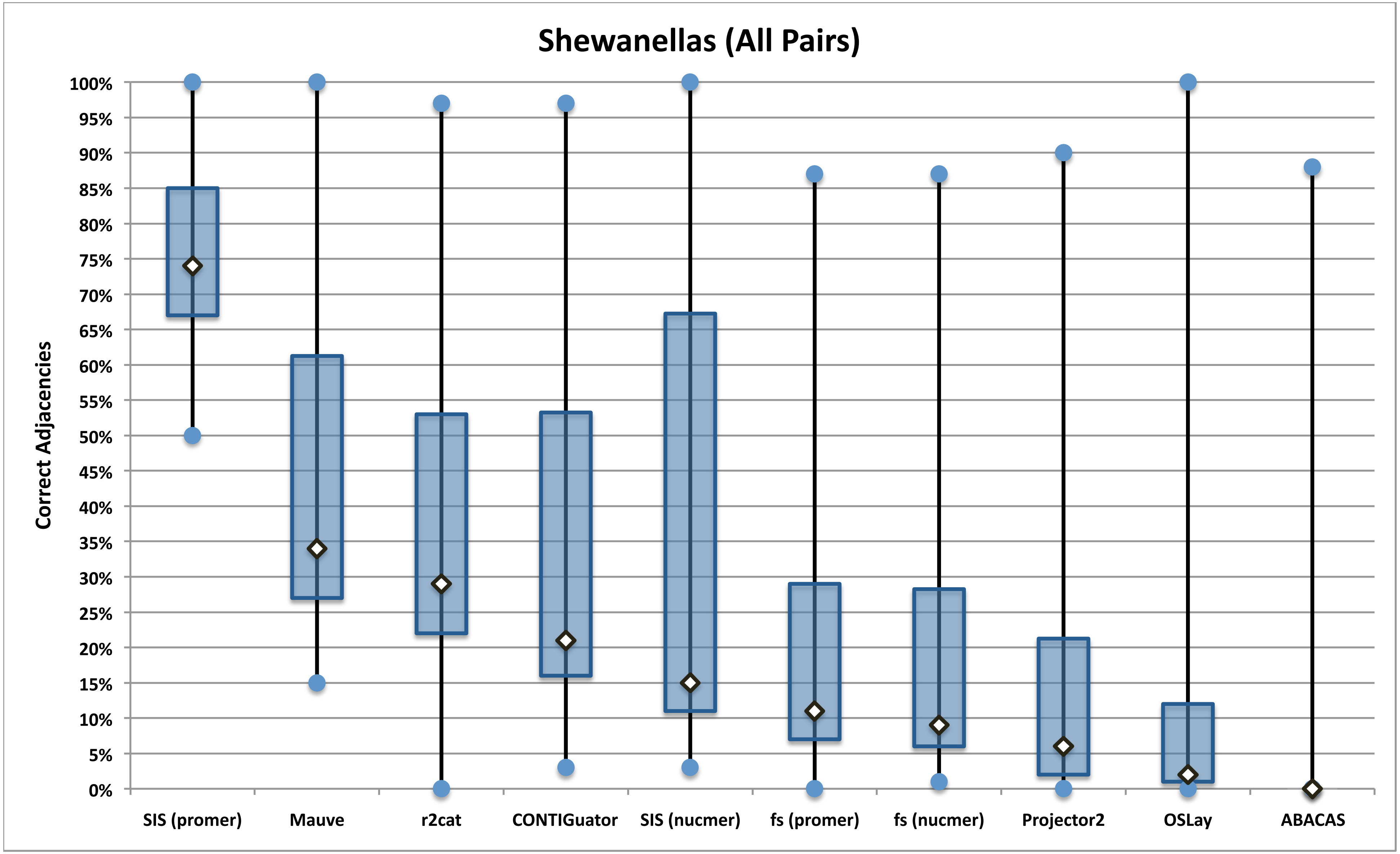

Shewanellas (All Pairs)
Correct Adjacencies
100%
95%
90%
85%
80%
75%
70%
65%
60%
55%
50%
45%
40%
35%
30%
25%
20%
15%
10%
5%
0%
SIS (promer)
Mauve
r2cat
CONTIGuator
SIS (nucmer)
fs (promer)
fs (nucmer)
Projector2
OSLay
ABACAS

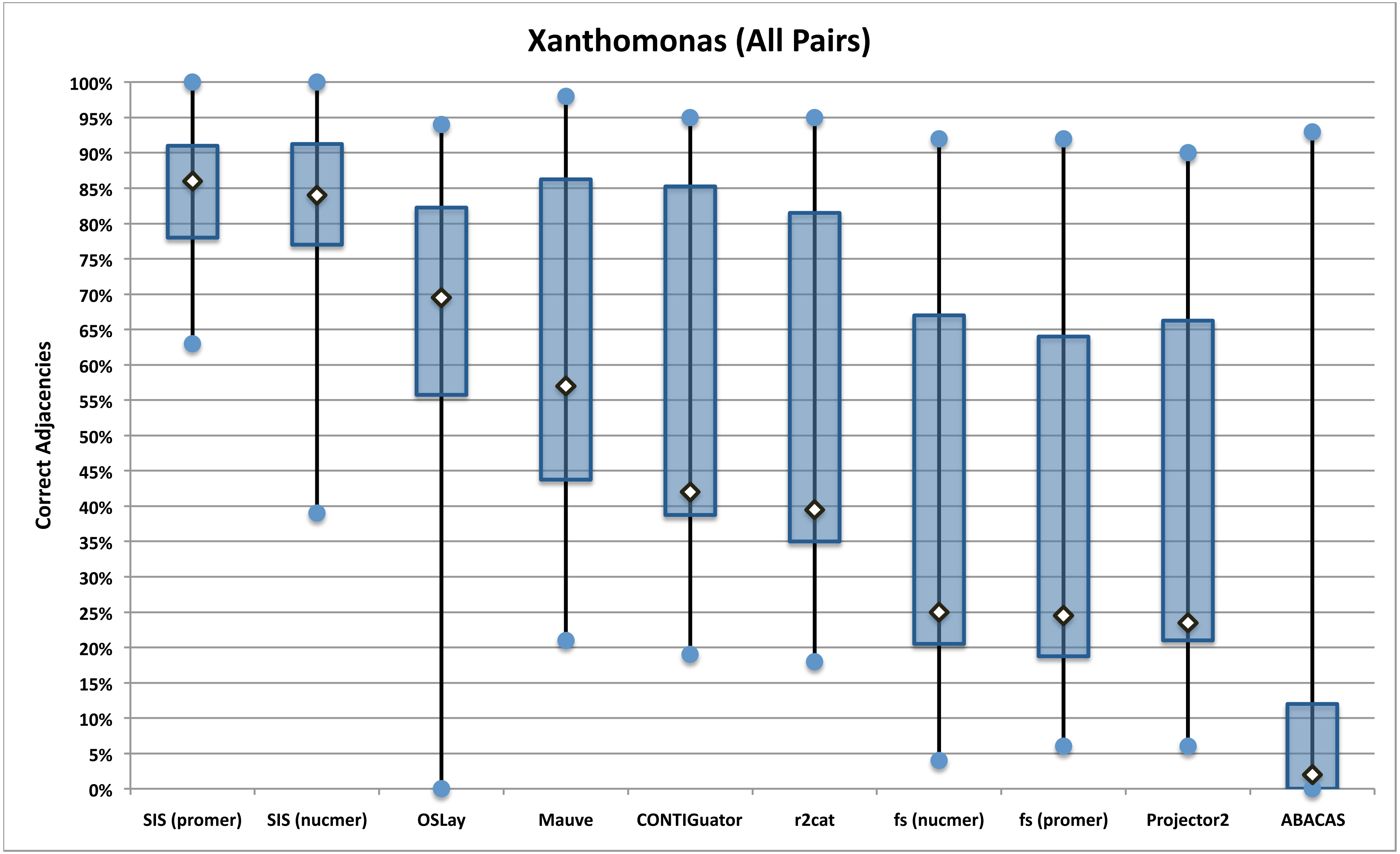

Xanthomonas (All Pairs)
Correct Adjacencies
100%
95%
90%
85%
80%
75%
70%
65%
60%
55%
50%
45%
40%
35%
30%
25%
20%
15%
10%
5%
0%
SIS (promer)
SIS (nucmer)
OSLay
Mauve
CONTIGuator
r2cat
fs (nucmer)
fs (promer)
Projector2
ABACAS

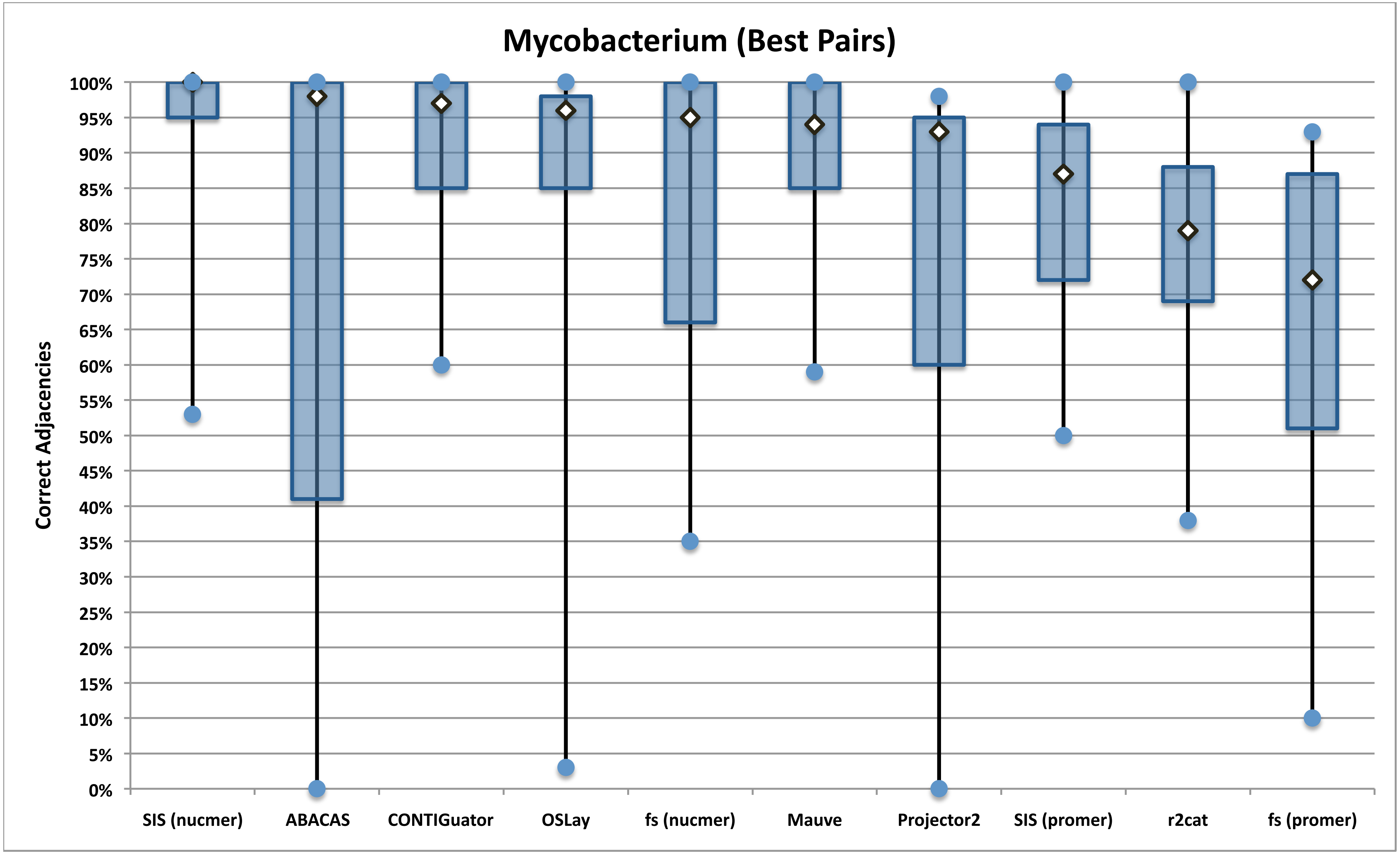

Mycobacterium (Best Pairs)
Correct Adjacencies
100%
95%
90%
85%
80%
75%
70%
65%
60%
55%
50%
45%
40%
35%
30%
25%
20%
15%
10%
5%
0%
SIS (nucmer)
ABACAS
CONTIGuator
OSLay
fs (nucmer)
Mauve
Projector2
SIS (promer)
r2cat
fs (promer)

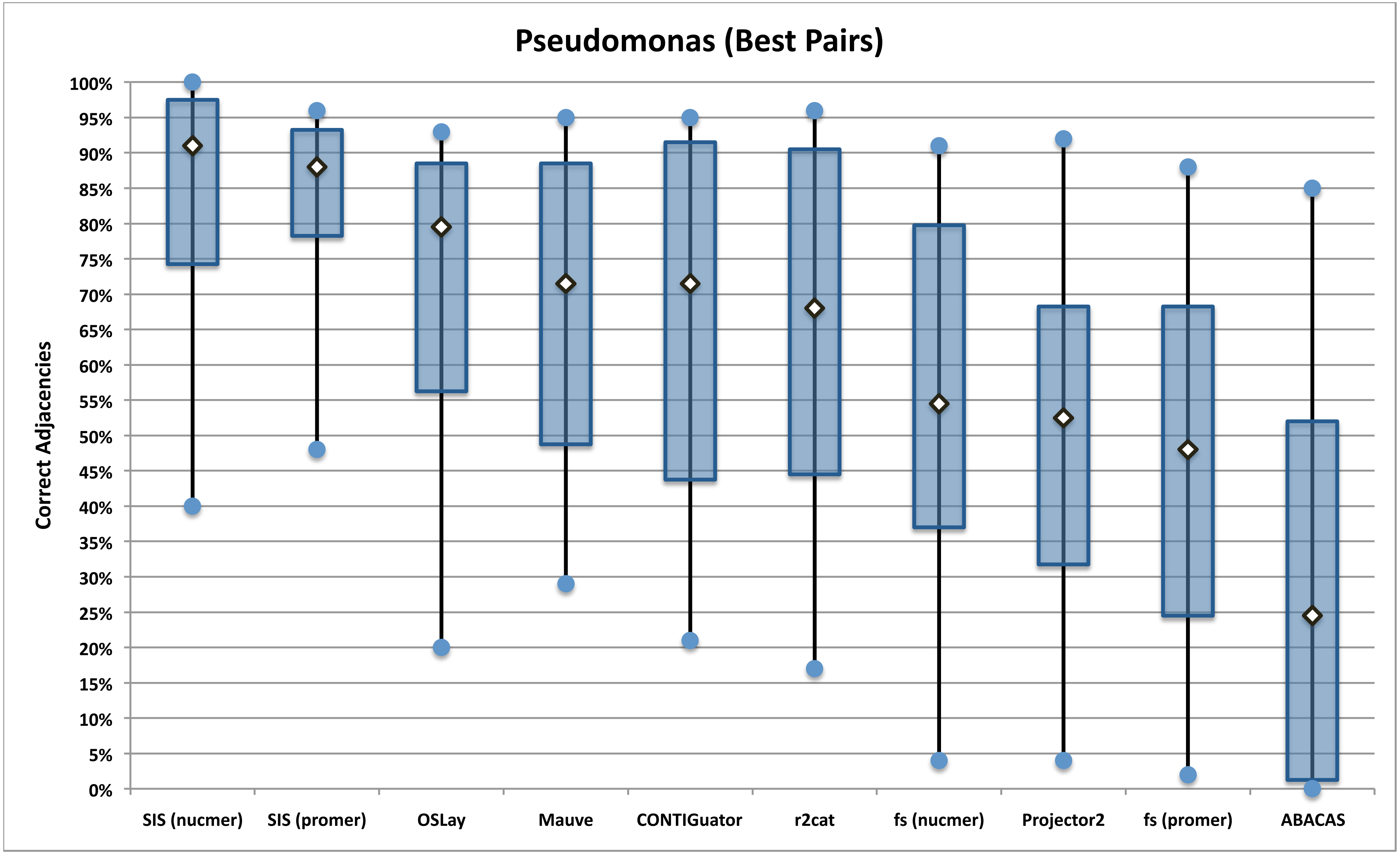

Pseudomonas (Best Pairs)
Correct Adjacencies
100%
95%
90%
85%
80%
75%
70%
65%
60%
55%
50%
45%
40%
35%
30%
25%
20%
15%
10%
5%
0%
SIS (nucmer)
SIS (promer)
OSLay
Mauve
CONTIGuator
r2cat
fs (nucmer)
Projector2
fs (promer)
ABACAS

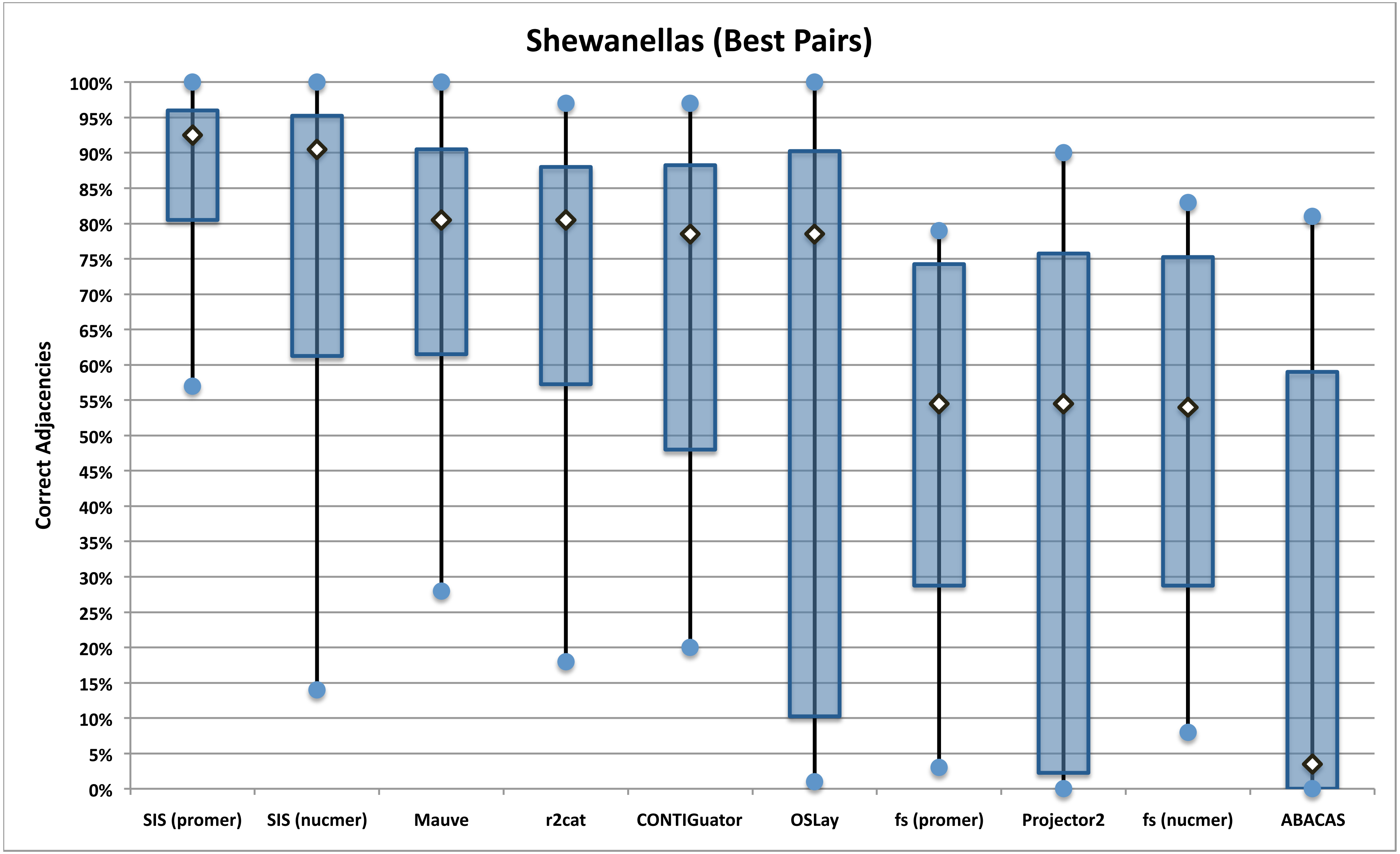
Shewanellas (Best Pairs)
Correct Adjacencies
100%
95%
90%
85%
80%
75%
70%
65%
60%
55%
50%
45%
40%
35%
30%
25%
20%
15%
10%
5%
0%
SIS (promer)
SIS (nucmer)
Mauve
r2cat
CONTIGuator
OSLay
fs (promer)
Projector2
fs (nucmer)
ABACAS

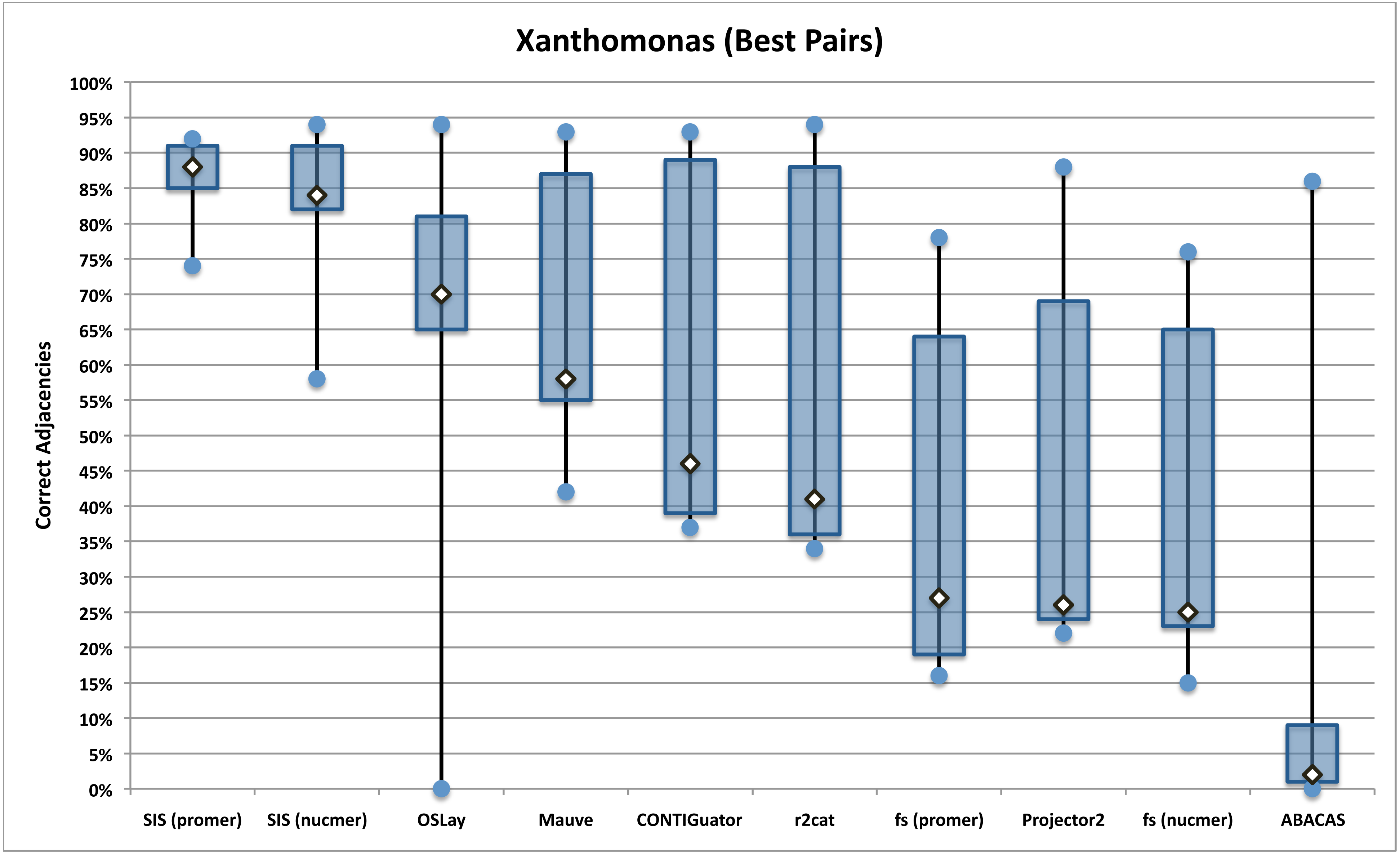

Xanthomonas (Best Pairs)
Correct Adjacencies
100%
95%
90%
85%
80%
75%
70%
65%
60%
55%
50%
45%
40%
35%
30%
25%
20%
15%
10%
5%
0%
SIS (promer)
SIS (nucmer)
OSLay
Mauve
CONTIGuator
r2cat
fs (promer)
Projector2
fs (nucmer)
ABACAS

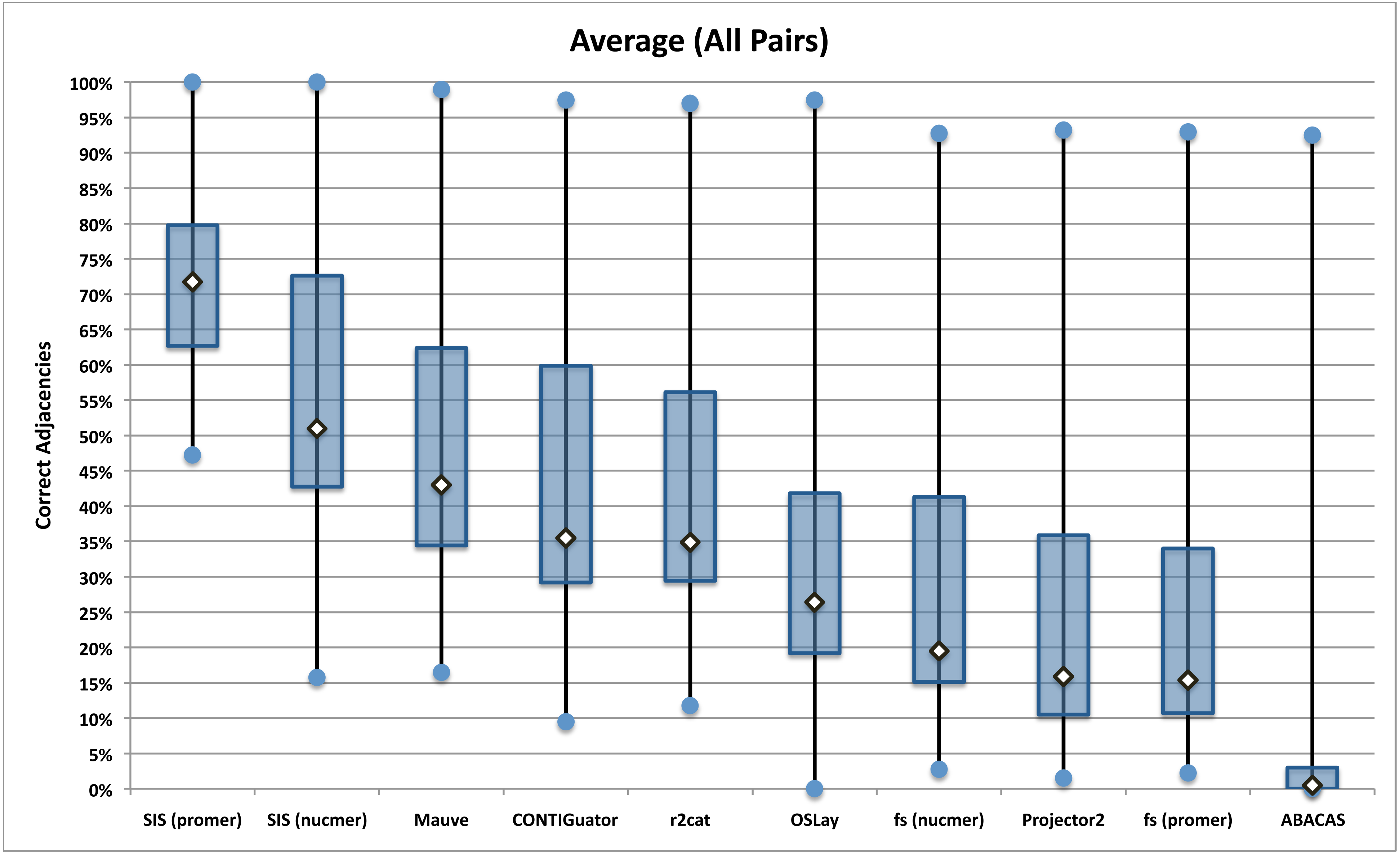

Average (All Pairs)
Correct Adjacencies
100%
95%
90%
85%
80%
75%
70%
65%
60%
55%
50%
45%
40%
35%
30%
25%
20%
15%
10%
5%
0%
SIS (promer)
SIS (nucmer)
Mauve
CONTIGuator
r2cat
OSLay
fs (nucmer)
Projector2
fs (promer)
ABACAS

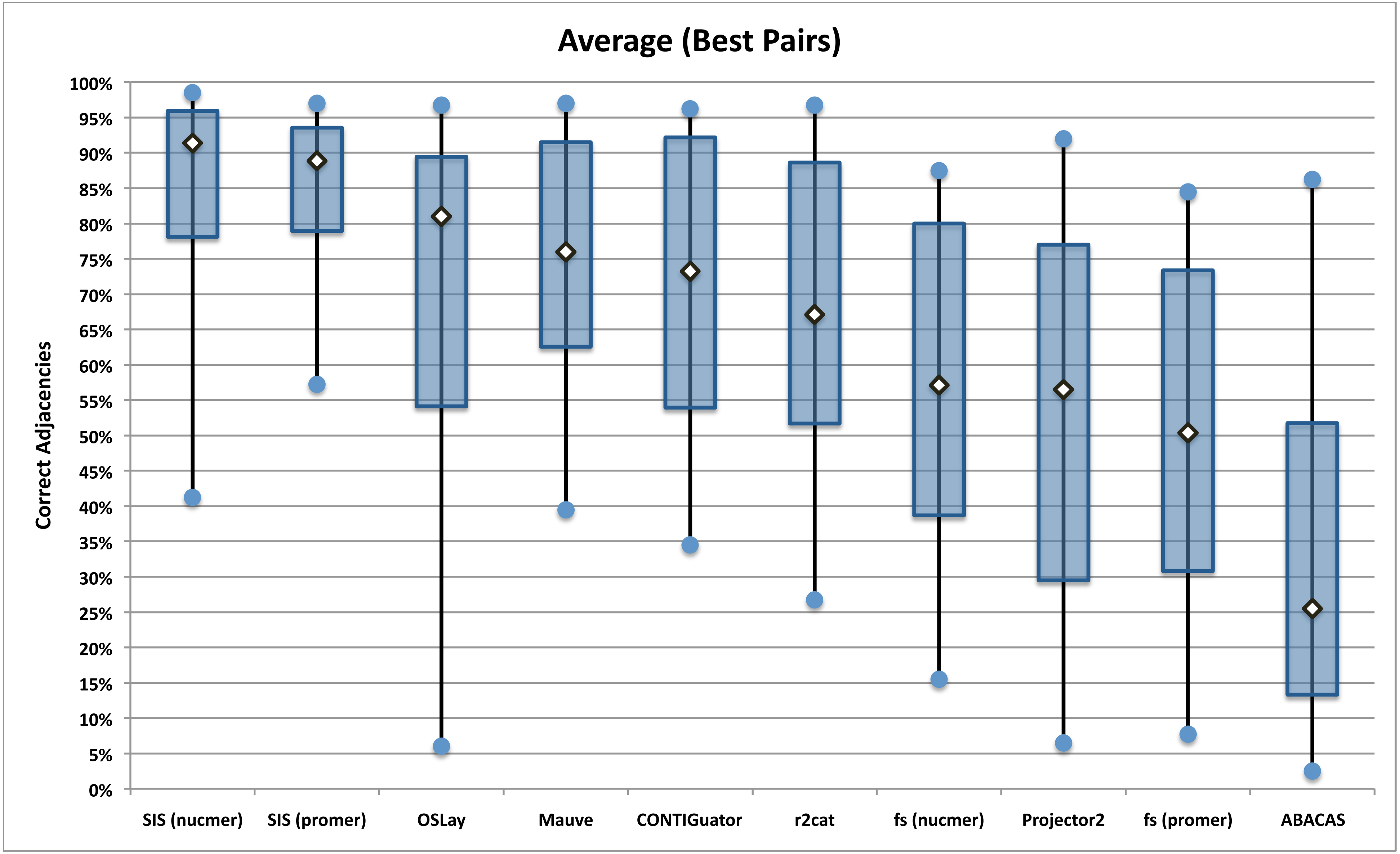

Average (Best Pairs)
Correct Adjacencies
100%
95%
90%
85%
80%
75%
70%
65%
60%
55%
50%
45%
40%
35%
30%
25%
20%
15%
10%
5%
0%
SIS (nucmer)
SIS (promer)
OSLay
Mauve
CONTIGuator
r2cat
fs (nucmer)
Projector2
fs (promer)
ABACAS
